# Characteristics of lower respiratory tract microbiota in the patients with post-hematopoietic stem cell transplantation pneumonia

**DOI:** 10.3389/fcimb.2022.943317

**Published:** 2022-09-13

**Authors:** Yukun He, Jia Li, Wenyi Yu, Yali Zheng, Donghong Yang, Yu Xu, Lili Zhao, Xinqian Ma, Pihua Gong, Zhancheng Gao

**Affiliations:** ^1^ Department of Respiratory and Critical Care Medicine, Peking University People's Hospital, Beijing, China; ^2^ Department of Respiratory, Critical Care, and Sleep Medicine, Xiang'an Hospital of Xiamen University, School of Medicine, Xiamen University, Xiamen, China

**Keywords:** hematopoietic stem cell transplantation, pneumonia, bronchoalveolar lavage, lower respiratory tract, microbiome

## Abstract

**Background:**

Pneumonia is a leading cause of non-relapse mortality after hematopoietic stem cell transplantation (HSCT), and the lower respiratory tract (LRT) microbiome has been proven to be associated with various respiratory diseases. However, little is known about the characteristics of the LRT microbiome in patients with post-HSCT compared to healthy controls (HC) and community-acquired pneumonia (CAP).

**Methods:**

Bronchoalveolar lavage samples from 55 patients with post-HSCT pneumonia, 44 patients with CAP, and 30 healthy volunteers were used to detect microbiota using 16S rRNA gene sequencing.

**Results:**

The diversity of the LRT microbiome significantly decreased in patients with post-HSCT pneumonia, and the overall community was different from the CAP and HC groups. At the phylum level, post-HSCT pneumonia samples had a high abundance of Actinobacteria and a relatively low abundance of Bacteroidetes. The same is true for non-survivors compared with survivors in patients with post-HSCT pneumonia. At the genus level, the abundances of *Pseudomonas*, *Acinetobacter*, *Burkholderia*, and *Mycobacterium* were prominent in the pneumonia group after HSCT. On the other hand, gut-associated bacteria, *Enterococcus* were more abundant in the non-survivors. Some pathways concerning amino acid and lipid metabolism were predicted to be altered in patients with post-HSCT pneumonia.

**Conclusions:**

Our results reveal that the LRT microbiome in patients with post-HSCT pneumonia differs from CAP patients and healthy controls, which could be associated with the outcome. The LRT microbiota could be a target for intervention during post-HSCT pneumonia.

## Introduction

Hematopoietic stem cell transplantation (HSCT) is a potentially curative method of treating hematologic and lymphoid malignancies (Harris et al., 2016). Overall survival following HSCT has significantly improved due to advances in transplant management (Gooley et al., 2010). The success of allogeneic HSCT, however, is hindered by certain complications (Bergeron and Cheng, 2017). Of all the organ-specific complications that can occur after HSCT, pneumonia is more complicated and difficult to treat and has been reported in 30-60% of HSCT recipients (Kader et al., 1994; Kotloff et al., 2004; Peters and Afessa, 2005; Lucena et al., 2014). Pneumonia, including community-acquired pneumonia (CAP) and hospital-acquired pneumonia (HAP), can occur early or late after the procedure, including during the pre-engraftment (neutropenic) phase and the early and late post-engraftment phases (Chi et al., 2013; Ahya, 2017; Bondeelle and Bergeron, 2019). Despite advances in posttransplant prevention support care, pneumonia remains a leading cause of non-relapse mortality after HSCT (Nusair et al., 2004; Choi et al., 2014; Harris et al., 2016; Zhou et al., 2019). A growing body of evidence suggests that gut microbiota is associated with pulmonary complications (PCs) after HSCT and transplant-related mortality (Taur et al., 2014; Harris et al., 2016; Golob et al., 2017), yet little is known about how lung microbiota is associated with disease status (Huang et al., 2013).

Advances in molecular methods and the advent of next-generation sequencing technologies have revealed that the lungs harbor complex and diverse bacterial communities (Charlson et al., 2011; Dickson et al., 2017; Huffnagle et al., 2017; Pattaroni et al., 2018). The potential role of the lung microbiome in respiratory pathology is increasingly being recognized (Dickson et al., 2016a; Invernizzi et al., 2020). Several studies have focused on defining lung microbiome composition in both healthy (Dickson et al., 2017) and diseased subsets (Dickson et al., 2014; Jorth et al., 2019; Singanayagam et al., 2019). The composition of the healthy lung microbiome may be depended on the neutral distribution of microbes from the oral cavity. Whereas microbiomes in diseased lungs may be associated strongly with increased selection of specific microbes, potentially reflecting possible microbiome alterations happening in its source environment at the same time (Hubbell, 2010; Venkataraman et al., 2015).

The balance of the lower respiratory tract (LRT) microbiome community in HSCT recipients is affected by multiple factors, including the impairment of host defenses through myeloablative conditioning, derangements in pulmonary immune responses, and multiple treatments, such as corticosteroid usage, and antibiotic usage (Gooley et al., 2010; Harris et al., 2016; Zinter and Hume, 2021), which collectively shape the respiratory microbiome. While the importance of the lung microbiome has already been proposed in the context of HSCT recipients with post-HSCT PCs (O’Dwyer et al., 2018), the characteristics of lung microbiota in HSCT recipients with pneumonia were less reported due to the complex conditions after HSCT. Moreover, the differences in lung microbiota related to the outcomes were undetermined. Therefore, we hypothesize that patients with post-HSCT pneumonia possessed lung microbiota that differs from that of healthy control subjects and CAP patients with normal immune function and lung microbiota could be differed in different clinical outcomes of patients with post-HSCT pneumonia.

To clarify the composition and putative function of the lung microbiota of patients with post-HSCT pneumonia, we applied 16S ribosomal ribonucleic acid (rRNA) gene sequencing to bronchoalveolar lavage fluid (BALF) samples from 55 patients with post-HSCT PCs, 44 patients with CAP, and 30 healthy control subjects (HCs).

## Materials and methods

### Study population

This study received ethics approval from the Ethical Review Committee of Peking University People’s Hospital (No. 2016PHB202-01). The study was performed in accordance with the Declaration of Helsinki. Written informed consent was obtained from all participants prior to clinical data collection and sampling.

A retrospective analysis was conducted for 55 patients with post-HSCT pneumonia (including CAP and HAP). Among them, patients who died after treatment were defined as non-survivors. Additionally, 30 healthy volunteers from the medical center were enrolled in the HC group voluntarily, and 44 immunocompetent CAP patients were recruited as disease control subjects. All participants were enrolled from Peking University People’s Hospital between April 2014 and August 2017. CAP and HAP were defined according to the published standards (Mandell et al., 2007; Kalil et al., 2016; Cao et al., 2018). Subjects with other pulmonary diseases, or tumors were excluded, and healthy volunteers taking antibiotics or hormones for the last three months were excluded (Charlson et al., 2011). General participant demographics, including age, gender, complications, laboratory findings, and clinical treatments, were collected from medical record system using a standard form. BALF was detected for routine microbiological examination, including routine culture, virus quantity polymerase chain reaction (qPCR), GeneXpert, 1,3-β-D glucan test, and galactomannan test. Baseline characteristics and clinical indicators of patients were listed in **Table 1** and **e-Tables 1-3**.

**Table 1:** Baseline characteristics and clinical indicators of patients with post-HSCT pneumonia, CAP patients, and health control subjects.

	post-HSCT pneumonia (N = 55)	CAP (N = 44)	HC (N = 30)	*P*-value
**Age**	34 (28-44.5)	53.5 (37-65.5)	60 (47.5-63.5)	<0.001 a^,b,^
**Gender, n, (% male)**	40 (72.7)	28 (63.6)	11 (36.7)	<0.001
**Smokers, n (%)**	4 (7.3)	8 (18.2)	0 (0)	0.009
**Comorbidities**				
**Diabetes Mellitus, n (%)**	6 (10.9)	6 (13.6)	3 (10.0)	0.234
**Hypertension, n (%)**	15 (32.7)	14 (31.8)	11 (36.7)	0.539
**Laboratory Findings**				
**Peripheral blood**				
**WBC (× 10^9/^L)**	4.31 (2.80-7.00)	6.40 (5.13-9.73)	6.87 (5.68-8.31)	<0.001 a^,b^
**Neutrophils (%)**	73.40 (59.80-86.30)	71.87 (63.02-83.02)	61.60 (53.72-64.77)	<0.001 a^,c^
**Lymphocytes (%)**	16.50 (7.70-27.1)	15.96 (8.92-27.19)	29.98 (24.93-36.83)	<0.001 a^,c^
**BAL related**				
**PMN percentages (%)**	14.00 (2.00-28.50)	18.00 (1.75-65.50)	1.00 (0.50-2.00)	<0.001 a^,c^
**Lymphocyte percentages (%)**	33.00 (17.00-53.50)	21.00 (11.00-43.50)	12.00 (8.00-30.75)	<0.001 a,
**Eosinophil percentages (%)**	0.00 (0.00-1.00)	0.00 (0.00-1.00)	0.00 (0.00-1.00)	0.042
**Macrophages percentages (%)**	42.00 (21.00-67.00)	37.00 (13.50-60.00)	86.50 (64.50-91.00)	<0.001 a^,c^
**Inflammatory markers**				
**PCT (μg/L)**	0.20 (0.11-0.50)	0.16 (0.05-1.02)	0.05 (0.05-0.09)	<0.001 a^,c^
**CRP (mg/L)**	30.09 (6.64-76.63)	40.75 (12.12-132.75)	1.37 (0.78-2.81)	<0.001 a^,c^
**ESR (mm)**	57.00 (27.75-89.00)	39.00 (19.00-61.00)	8.50 (6.00-13.50)	<0.001 a^,c^
**PSI**	74 (65-88)	72 (51-104)	–	0.235

a, HSCTvs.HC; b, HSCTvs.CAP; c, CAPvs.HC; HSCT hematopoietic stem cell transplantation; CAP community-acquired pneumonia; CRP, C-reactive protein; ESR, erythrocyte sedimentation rate; PCT procalcitonin; PSI, pneumonia severity index; PMN, polymorphonuclear leukocyte; WBC, white blood cell.

### Sample preparation, DNA extraction, and sequencing

For patients diagnosed with pneumonia, bronchoscopy was performed as a part of clinical management within 72 h after hospital admission before the ventilation treatments. Detailed sampling procedures, pretreatment, and storage were performed as described in previous studies (Zheng et al., 2019; He et al., 2022). Sampling controls were collected through simulated bronchoscopy procedures (no patient) using the same instrument and method. BALF was then centrifuged. Total DNA was extracted from the BALF precipitate and 500 µL of the supernatant using the CTAB/SDS method. 16S rRNA genes of the V3‐V4 region were amplified with pre-validated primers (Klindworth et al., 2013). Detailed procedure of library construction was provided in the supplementary materials. High‐quality libraries were sequenced to 250 bp paired-end raw reads on the HiSeq2500 platform. The V3-V4 regions were not amplified in the sampling control. Besides, contaminants were excluded from this analysis using negative controls containing only sterile ddH_2_O and reagents for extraction and PCR amplification. “Decontam” R package was applied to further reduce the potential impact of contamination for low biomass samples using the prevalence method in the “isNotContaminant” function with thresholds at 0.1 (Davis et al., 2018; Karstens et al., 2019) (**e-Table 4**). While all the steps of sampling, DNA extraction and PCR amplification were controlled with negative reagents, further analysis was performed to ensure that the potential risks of contamination were minimized. The results of this study were compared with the 92 contamination genera detected in the negative sequencing blank controls in previous study (Salter et al., 2014). We failed to detect 61 out of the contaminant genera in our result (**e-Table 5**). Among the remaining 31 genera found in our data, 6 genera were not reported in samples from the respiratory tract but none had an average relative abundance greater than 0.0002.

### Bioinformatics analysis

The high-quality sequencing data was generated by removing low-quality reads (the quality control rate is less than 1%), primers, barcodes, and dereplication using VSEARCH (Rognes et al., 2016). After removing the chimeric sequences with the UCHIME algorithm (Edgar et al., 2011), effective reads were obtained and denoised to generate amplicon sequence variants (ASVs) using unoise3 (Edgar, 2016). Taxonomy assignment was performed on ASVs based on the RDP database (v 11.5) (Cole et al., 2014) and the GreenGene database (DeSantis et al., 2006).The microbiome phenotypes were predicted by BugBase based on GreenGene annotation (Ward et al., 2017). PICRUSt2 was used to identify the predicted associated pathways from the inferred metagenomes of taxa with the ‘stratified’ mode (Douglas et al., 2020).

### Statistical analysis

The abundance-based coverage estimator (ACE) index and Shannon index were calculated to evaluate alpha diversity using rarefied data using vegan R package (Jari Oksanen et al., 2020). The nearest taxon index (NTI) and net-relatedness index (NRI) were applied to estimate phylogenetic structure of the community using picante R package (Kembel et al., 2010). The algorithm was run using 999 randomizations of the community within the mega-phylogeny applying the “taxa.labels”. Beta diversity was assessed by permutational multivariate analysis of variance (PERMANOVA) test, and visualized using principal coordinate analysis (PCoA). Adonis test was conducted based on Bray-Curtis distances and Jaccard distances. ASV abundances were centered with log-ratio transformation prior to analysis. Differential bacterial taxa among groups were assessed using the edgeR R package based on centered log-ratio-transformed genome relative abundance (Robinson et al., 2010). A multifactorial design within the edgeR R package was used to adjust confounding factors, such as age, gender, and smoking. Statistically significant genera differences (LDA > 2, P < 0.05) associated with different groups were explored using linear discriminant analysis (LDA) effect size (LEfSe) (Segata et al., 2011). Spearman’s rho was calculated using the ‘corr.test’ function within the R package. Network analysis was performed based on read count data at the genus level using SpeciEasi (Kurtz et al., 2015). The density of the networks was calculated by the “graph.density” function in the igraph R package.

For clinical indicators, all categorical variables are presented as numbers (percentages), parametric continuous variables are presented as the mean ± SD, and nonparametric continuous variables are presented as median and interquartile ranges (25th and 75th percentiles). Student’s t-test or analysis of variance (ANOVA) with *post hoc* Tukey HSD test were used to analyze continuous parametric data. Continuous nonparametric data were analyzed using Mann–Whitney U or a Kruskal–Wallis test. All categorical data were analyzed using a chi-square or Fisher’s exact test. All tests were two-sided, p-values were corrected using Benjamin-Hochberg false discovery rate (FDR), and p < 0.05 was considered statistically significant. Statistical analyses were performed using SPSS version 23 software.

## Results

### Clinical characteristics of the study population

To explore the clinical characteristics of post-HSCT pneumonia, we summarized clinical information from 55 subjects. For HSCT patients with pneumonia, the median age was 34 years old, and 72.7% of them were male. A total of 47.3% of patients underwent transplantation for acute myelocytic leukemia (AML). **e-Table 1** showed that 18 (32.7%) patients died. Pneumonia occurred at late post-engraftment phases in 35 (63.6%) patients, and occurred at pre-engraftment (neutropenic) phase in 4 (7.3%) patients (**e-Table 1**). The median time from transplant to the onset of pneumonia was 165 days. The PMN percentages in the BALF and PCT were significantly elevated in non-survivors (**e-Table 2**), suggesting that exacerbated inflammation may result in poor outcomes in patients with post-HSCT pneumonia.

Demographic information on the three groups is displayed in **Table 1**, along with comorbidities, blood cell counts, BALF cell counts, inflammatory markers, and the pneumonia severity index (PSI). Neutrophil percentage, C-reactive protein (CRP), procalcitonin (PCT) and erythrocyte sedimentation rate (ESR) levels, and the percentage of polymorphonuclear leukocytes (PMNs) in the BALF were higher in patients with pneumonia than in the HC group. In contrast, the lymphocyte percentage in the peripheral blood and the macrophage percentage in the BALF were significantly lower. Pathogens were detected in 47.3% of BALF samples with conventional hospital-based microbiology tests in patients with post-HSCT pneumonia and 65.9% in patients with CAP (**e-Table 3**). 89.1%, 81.8%, and 54.5% patients with post-HSCT pneumonia received the antibiotics, antifungals, and *Pneumocystis carinii* prophylaxis, respectively (**e-Table 3**). Moreover, there was no difference in the proportion of patients taking antibiotics in the last three months between the two diseased groups (**e-Table 3**). and the mortality is higher in the post-HSCT pneumonia group.

### Disturbance of the LRT microbiome

**Figure 1:**
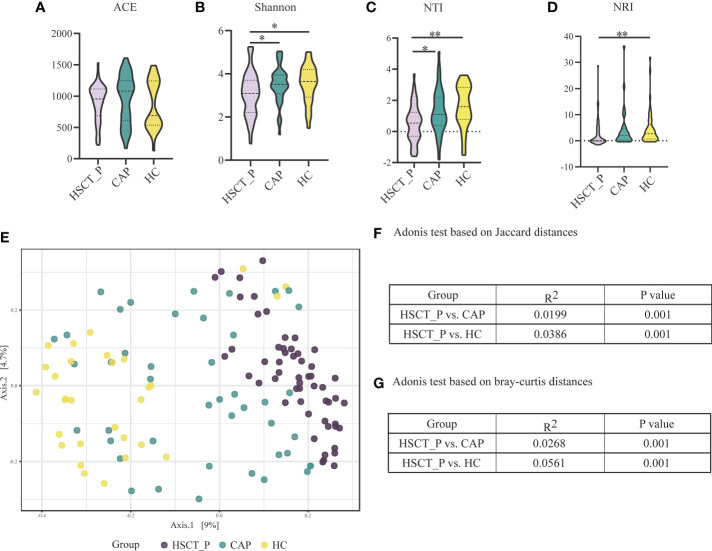
Alpha and Beta diversity of the lower respiratory tract microbiome in the post-HSCT pneumonia (HSCT_P), community-acquired pneumonia (CAP), and healthy controls (HC) groups. **(A)** Comparison of abundance-based coverage estimator (ACE) index in different groups for assessment of microbiome richness of three groups. **(B)** Comparison of Shannon index in different groups for assessment of microbiome diversity of three groups. * represents *p* < 0.05 based on Kruskal–Wallis test. **(C)** Comparison of the mean nearest taxon distance (MNTD) index in different groups for assessment of phylogenetic diversity of three groups. ** represents *p* < 0.01 based on Kruskal–Wallis test. **(D)** Comparison of the nearest taxon index (NTI) in different groups for assessment of microbiome diversity of three groups. * represents *p* < 0.05, and ** represents *p* < 0.0 based on Kruskal–Wallis test. **(E)** Beta diversity was assessed by PERMANOVA test based on Jaccard distances using principal coordinate analysis (PCoA). *P* value of post-HSCT pneumonia vs. CAP and post-HSCT pneumonia vs. HC were both 0.001. **(F)** Adonis test based on Jaccard distances. **(G)** Adonis test based on bray-curtis distances.

Species accumulation curves and the read counts of samples revealed that the sequencing was sufficient to describe associated microbial community (**e-Figure 1**; **e-Table 6**). After filtering for sequence variants in at least two samples with a minimum relative abundance of 0.05% (Bowerman et al., 2020), 2396 sequence variants were retained for community analysis. In terms of alpha diversity, no significant differences were observed in the ACE index, whereas the Shannon index showed significant decreases in diversity in patients with post-HSCT pneumonia compared to the HC and CAP groups (**Figures 1A, B**). The mean values of NTI and NRI in all groups was greater than 0, indicating the samples clustering in each group (**Figures 1C, D**). The NTI values in samples with post-HSCT pneumonia were less positive compared to the CAP and HC samples, which indicated that phylogenetic clustering was weakest in the post-HSCT pneumonia samples. The PERMANOVA test demonstrated that the bacterial community of the post-HSCT pneumonia group significantly differs from that of the CAP or HC group (**Figure 1E**). The Adonis tests based on Bray-Curtis distances and Jaccard distances revealed that significant differences existed in both composition and abundance of tax among groups (**Figures 1F, G**). Besides, we found no significant difference in the microbiota composition in terms of smoking status (*p* = 0.297) in all subjects and in the HC group, there is no difference in sex (*p* = 0.19), age (*p* = 0.052) or comorbidity groups. Similarly, antibiotic usage (*p* = 0.278) or corticosteroid usage (*p* = 0.552) had no effect in the microbiota composition of patients (**e-Figures 2A–G**). These results indicate that patients with post-HSCT pneumonia had decreased diversity and have a different LRT microbiome composition compared with the HC or CAP group.

**Figure 2:**
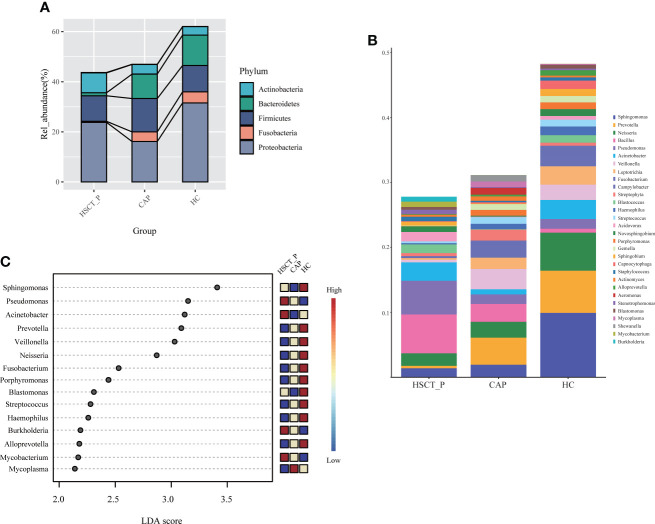
Taxonomic analysis of lower respiratory tract microbiome in the post-HSCT pneumonia (HSCT_P), community-acquired pneumonia (CAP), and healthy controls (HC) groups. **(A)** The relative abundance of microbial communities at the level of phylum among groups. **(B)** The top 30 genera in three groups. **(C)** LDA shows distinct lung microbiome composition associated with HSCT_P, CAP, and HC group. LDA scores as calculated by LEfSe of taxa differentially abundant in different group.

To determine specific bacterial taxa correlated with patients with post-HSCT pneumonia, we compared the relative abundances (RA) of microbiota among groups. As shown in **Figure 2A**, the five most abundant phyla included Proteobacteria, Firmicutes, Bacteroidetes, Fusobacteria, and Actinobacteria. Specifically, post-HSCT pneumonia samples had a higher RA of Actinobacteria and a relatively low RA of Fusobacteria and Bacteroidetes. The top 30 genera in RA were shown in **Figure 2B**. Obviously, the RA of *Sphingomonas*, and *prevotella* decreased in the post-HSCT pneumonia group (**Figure 2B**; **e-Tables 7, 8**), whereas the RA of *Bacillus*, *Bifidobacterium*, and *Enterococcus* were significantly increased. According to the Lefse analysis, the RA of *Pseudomonas*, *Acinetobacter*, *Burkholderia*, and *Mycobacterium* were prominent in the pneumonia group after HSCT (**Figure 2C**).

### Potential function of the LRT microbiome

Predicted phenotypes based on taxonomic classification were analyzed with BugBase, which indicated that aerobic bacteria were more abundant in patients with post-HSCT pneumonia than in the CAP and HC groups, while the abundance of anaerobic bacteria was the opposite (**e-Figures 3A, B**, *p* < 0.05). Additionally, the results suggested that Gram-positive bacteria were more abundant in both post-HSCT pneumonia and CAP groups than in the HC group, while the abundance of Gram-negative bacteria was lower (**e-Figures 3C, D**, *p* < 0.05).

**Figure 3:**
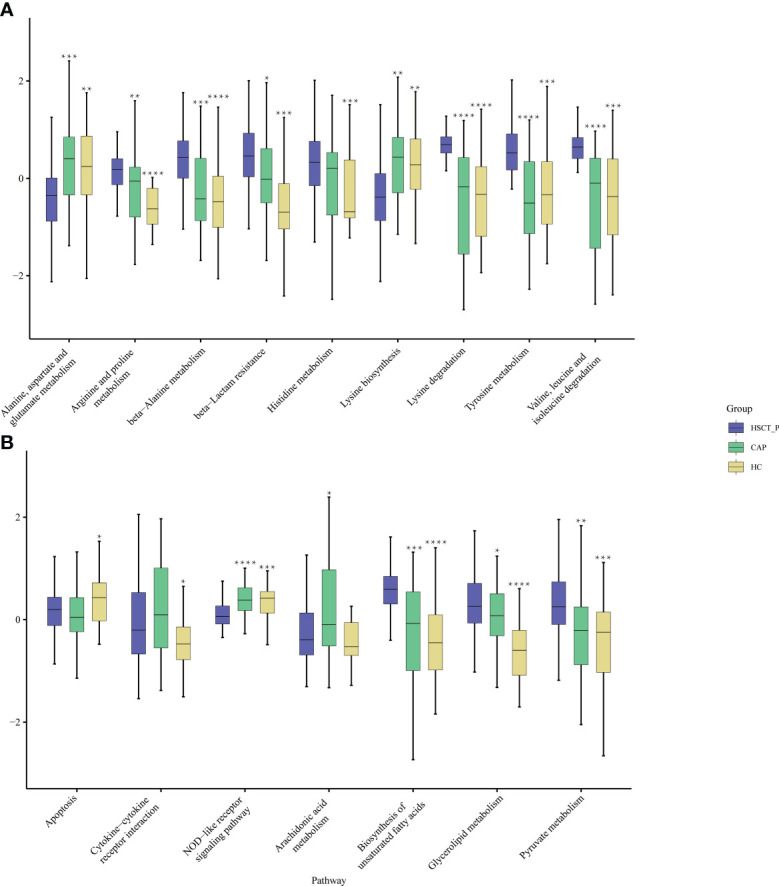
Functional characterization of different groups based on PICRUSt analysis. The abundance of pathways concerning amino acid metabolism among three groups **(A)** and the abundance of pathways concerning lipid metabolism and immune reaction among three groups **(B)**. Black stars upon the boxes indicate significant results for CAP or group compared with post-HSCT pneumonia patients. (* P < 0.05, ** P < 0.01, *** P<0.001, ****P < 0.0001).

To explore differences in potential function, we annotated the 16S reads using PICRUSt2 based on the Kyoto Encyclopedia of Genes and Genomes (KEGG) (Kanehisa et al., 2021), and obtained 193 KEGG pathways. Through a Wilcoxon Rank Sum Test, we found that 99 and 107 pathways may be differentially expressed in the post-HSCT pneumonia group compared with CAP and HC groups, respectively (**e-Tables 9, 10**). The expression of multiple pathways concerning amino acid metabolism, such as arginine, histidine, and tyrosine, was elevated in patients with post-HSCT pneumonia compared to the two control groups (**Figure 3A**). The pathways “biosynthesis of unsaturated fatty acids” and “pyruvate metabolism” were predicted to be more enriched in patients with post-HSCT pneumonia (**Figure 3B**). Compared with the HC group, pathways associated with “cytokine-cytokine receptor interaction” were more abundant in patients with post-HSCT pneumonia, but the predicted “NOD-like receptor signaling” pathway decreased (**Figure 3B**). These results suggest that the predicted microbial functions of amino acids, lipid metabolism, and inflammatory reactions may change in the patients with post-HSCT pneumonia.

### The LRT microbiome in non-survivors

**Figure 4:**
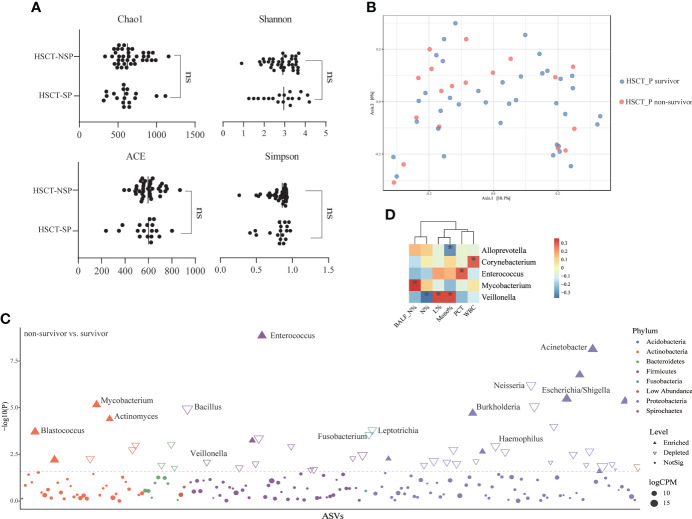
Differences in lung microbial composition between survivors and non-survivors of post HSCT pneumonia patients **(A)** Comparison of Chao1, ACE, Shannon and Simpson diversity index in different groups for assessment of microbiome alpha diversity of survivors and non-survivors. ns, no significant differences were observed between the groups. **(B)** Beta diversity was assessed by PERMANOVA test based on Jaccard distances using principal coordinate analysis (PCoA) (*p* = 0.361). **(C)** Differential genera between survivors and non-survivors using edgeR package. CPM, counts per million. **(D)** Spearman’s rho calculated between ASVs and clinical indicators. Black stars within heatmap boxes indicate significant results (*p < 0.05), Benjamini–Hochberg adjustment for multiple comparisons. ASV abundances were centered with log-ratio transformation prior to analysis.

To explore the structure of the flora in patients with post-HSCT pneumonia, we compared subgroups within the 55 samples. No significant difference was observed in microbiota composition or diversity in terms of pathogen detection (**e-Figure 4**) or post-HSCT period (neutropenic phase, early and late post-engraftment phase, **e-Figure 5**) or outcome (survivors vs. non-survivors) (**Figures 4A, B**). However, a higher RA of Actinobacteria and a lower RA of Bacteroidetes were found in non-survivors (**e-Figure 6**), and 45 genera were identified as differential taxa between survivors and non-survivors (**e-Table 11**). The RAs of the genera *Enterococcus*, *Acinetobacter*, *Burkholderia*, *Mycobacterium*, and *Escherichia* all significantly increased in non-survivors, while the RAs of the genera *Neisseria*, *Bacillus*, and *Veillonella* decreased in non-survivors (**Figure 4C**). Among them, the RA of *Enterococcus* was positively correlated with PCT levels, and *Mycobacterium* was positively correlated with PMN percentage in the BALF numerically (**Figure 4D**; **e-Table 12**). The RA of *Veillonella* was negatively correlated with the neutrophil percentage. Through phenotype prediction, potentially pathogenic and aerobic bacteria were enriched in non-survivors (**e-Figure 7**, all *p* < 0.05).

## Discussion

The LRT microbiota is crucial for the host immune system, and the imbalance between microbial migration and removal is correlated with alveolar and systematic inflammation (O’Dwyer et al., 2016). Understanding the composition of the LRT microbiota under different diseases is essential for identifying potential mechanisms underlying the pathogenesis of post-HSCT pneumonia and could offer crucial insights into therapeutic targets. Although sputum sample is noninvasive and was used to explore the microbiome in diverse pulmonary diseases, it is difficult to collect for some patients and all healthy controls. Besides, the location of the respiratory tract represented by the sputum sample cannot be determined and sputum sample is highly likely to be contaminated by the oral microbiota. On the contrary, BALF has been believed to be a viable option for the lung microbiome (Cheng et al., 2020). Thus, in this study, we characterized the LRT microbiome of 55 patients with post-HSCT pneumonia and provided evidence for significant alterations in the bacterial community, utilizing the remaining BALF samples from clinical testing.

Previous studies have found that intestinal microbiota and dental biofilm microbiota dysbiosis occurred during HSCT, marked by a gradual loss of bacterial diversity (Heidrich et al., 2021). Our results also showed a significantly decreased alpha diversity in patients with post-HSCT pneumonia compared to the CAP and HC groups. The composition of the LRT microbiome in post-HSCT pneumonia patients was characterized by a decreased abundance of commensal genera and an overgrowth of opportunistic pathogens, such as *Acinetobacter* (Du et al., 2021) and *Mycobacterium* (Meehan et al., 2021). Thus, under the state of immune defects and chronic inflammation, the bacterial community was likely to be seriously altered in patients after HSCT, which elicited epithelial and luminal inflammation, which may further alter the conditions in the lung microenvironment, perpetuating dysbiosis and increasing the susceptibility to infection. However, although Nearing et al. reported that edgeR and LEfSe often identified the most significant differential ASVs than any other tools, they have relatively high false discovery rate (Nearing et al., 2022).

According to the predicted phenotypes, the abundance of anaerobic bacteria decreased in patients with post-HSCT pneumonia, and at the phylum level, a relatively low abundance of Bacteroidetes was observed compared with the HC group. The same results were also observed in non-survivors of the post-HSCT pneumonia group compared to the survivors. Marsland et al. reported that Bacteroidetes are strictly anaerobic and that most Bacteroidetes are sensitive to low pH (Marsland and Gollwitzer, 2014). Robustly disturbed and reduced anaerobic bacteria could be caused by chemotherapy, antibiotics, mechanical ventilation, or HSCT itself, and inflammatory response could lower the local PH in the LRT. Antibiotics targeting anaerobic pathogens have been revealed to increase GVHD-related mortality in humans (Shono et al., 2016). Therefore, dysbiosis and the decrease of anaerobic bacteria in the LRT could be the cause of post-HSCT pneumonia and poor prognosis. However, the exact mechanisms must be further studied.

The predicted functions of the LRT microbiome were significantly different among groups. The relative abundances of microbiome genes associated with histidine metabolism were increased in patients with post-HSCT pneumonia, and the bioavailable histidine in the lung could promote *Acinetobacter* pathogenesis and serve as a crucial nitrogen source during infection (Lonergan et al., 2020). The “cytokine-cytokine receptor interaction” function was predicted to be more evident in patients with post-HSCT pneumonia, but the abundance of genes related to “NOD-like receptor signaling” decreased. This may indicate a stronger inflammatory response and impaired innate immune responses in the LRT of patients with post-HSCT pneumonia. However, the predictive ability of PICRUSt2 is limited and the actual functions can substantially differ. Meanwhile, inaccuracies in pathway annotation or assignments of gene function may be present. Thus, further experiments are required to verify changes in these functions.

Our data further demonstrated that some genera were correlated with the prognosis of patients with post-HSCT pneumonia. The RA of *Enterococcus* was more abundant in non-survivors, which agrees with recent studies reporting that the enrichment of gut-associated bacteria in the lung suggested poor outcomes for critical patients (Dickson et al., 2016b; Dickson et al., 2020; Martin-Loeches et al., 2020). This suggests that increased intestinal permeability is involved in the gut-lung translocation of bacteria and inflammatory products to distant organs in non-survivors, which should be investigated further using paired gut and lung specimens.

There are some limitations to our study. First, clinical heterogeneity is a major concern in patients with post-HSCT pneumonia (including the primary disease, pretreatment methods, and transplant types). Meanwhile, nearly all HSCT recipients were received therapy such as antibiotics, corticosteroids and cytotoxic drugs inevitably, so the lung microbiome in the post-HSCT pneumonia group was shaped by a variety of factors. Thus, the direct cause of the differences in the lung microbiome between the post-HSCT pneumonia group and HC group is unclear. Therefore, further experiment is needed to explore lung microbiome characteristics in this population and the effects of each variability. Second, the causal relationship between the observed alterations in the LRT microbiome and the development of post-HSCT pneumonia is uncertain, which should be further explored using animal models. Third, the 16s resolution is lower, and only relative abundances of specific bacteria were described, meaning that further research to detection of absolute abundances of microorganisms are needed. Lastly, despite the efforts made in contamination control, the potential sources of oral or environmental pollution may be not completely excluded.

Despite these limitations, our study explored the LRT microbiome in patients with post-HSCT pneumonia and raises some interesting questions worthy of further investigation. Our results support the findings of larger cohorts evaluating the value of the airway microbiome and its immune interactions and propose potential targets for preventing and treating pneumonia in post-HSCT patients.

## Conclusions

Our results demonstrate that the LRT microbiome in post-HSCT pneumonia, which is characterized by decreases in species diversity, the enrichment of pathogens, and reduced biotic interactions, differs from CAP patients and healthy controls. The composition of the LRT microbiome is different with outcomes in patients with post-HSCT pneumonia.

## Data availability statement

The names of the repository/repositories and accession number(s) can be found below: https://www.ncbi.nlm.nih.gov/, PRJNA791752 and PRJNA751994.

## Ethics statement

The studies involving human participants were reviewed and approved by the Ethical Review Committee of Peking University People’s Hospital. The patients/participants provided their written informed consent to participate in this study.

## Author contributions

YH was responsible for investigation, methodology, analyzation, and writing-original draft. JL was responsible for investigation, methodology, analyzation. WY, YZ, DY, YX, LZ, and XM were responsible data collection, experiment and analyzation. PG and ZG were responsible for investigation, methodology, provision of in-house control data, project administration, resources, supervision, funding acquisition, writing-review and editing. All authors read and approved the final manuscript.

## Funding

This work was supported by the National Natural Science Foundation of China (No. 82000019, No. 81870010), and the National and Provincial Key Clinical Specialty Capacity Building Project 2020 (Department of the Respiratory Medicine).

## Acknowledgments

The authors wish to thank staff members of the hospital for assistance with samples and clinical data collection.

## Conflict of interest

The authors declare that the research was conducted in the absence of any commercial or financial relationships that could be construed as a potential conflict of interest.

## Publisher’s note

All claims expressed in this article are solely those of the authors and do not necessarily represent those of their affiliated organizations, or those of the publisher, the editors and the reviewers. Any product that may be evaluated in this article, or claim that may be made by its manufacturer, is not guaranteed or endorsed by the publisher.

## References

[B1] Ahya V. N. (2017). Noninfectious acute lung injury syndromes early after hematopoietic stem cell transplantation. Clin. Chest Med. 38 (4), 595–606. doi: 10.1016/j.ccm.2017.07.002 29128012 PMC7131710

[B2] Bergeron A. Cheng G. S. (2017). Bronchiolitis obliterans syndrome and other late pulmonary complications after allogeneic hematopoietic stem cell transplantation. Clin. Chest Med. 38 (4), 607–621. doi: 10.1016/j.ccm.2017.07.003 29128013

[B3] Bondeelle L. Bergeron A. (2019). Managing pulmonary complications in allogeneic hematopoietic stem cell transplantation. Expert Rev. Respir. Med. 13 (1), 105–119. doi: 10.1080/17476348.2019.1557049 30523731

[B4] Bowerman K. L. Rehman S. F. Vaughan A. Lachner N. Budden K. F. Kim R. Y. . (2020). Disease-associated gut microbiome and metabolome changes in patients with chronic obstructive pulmonary disease. Nat. Commun. 11 (1), 5886. doi: 10.1038/s41467-020-19701-0 33208745 PMC7676259

[B5] Cao B. Huang Y. She D. Y. Cheng Q. J. Fan H. Tian X. L. . (2018). Diagnosis and treatment of community-acquired pneumonia in adults: 2016 clinical practice guidelines by the Chinese thoracic society, Chinese medical association. Clin. Respir. J. 12 (4), 1320–1360. doi: 10.1111/crj.12674 28756639 PMC7162259

[B6] Charlson E. S. Bittinger K. Haas A. R. Fitzgerald A. S. Frank I. Yadav A. . (2011). Topographical continuity of bacterial populations in the healthy human respiratory tract. Am. J. Respir. Crit. Care Med. 184 (8), 957–963. doi: 10.1164/rccm.201104-0655OC 21680950 PMC3208663

[B7] Cheng C. Wang Z. Wang J. Ding C. Sun C. Liu P. . (2020). Characterization of the lung microbiome and exploration of potential bacterial biomarkers for lung cancer. Transl. Lung Cancer Res. 9 (3), 693–704. doi: 10.21037/tlcr-19-590 32676331 PMC7354118

[B8] Chi A. K. Soubani A. O. White A. C. Miller K. B. (2013). An update on pulmonary complications of hematopoietic stem cell transplantation. Chest 144 (6), 1913–1922. doi: 10.1378/chest.12-1708 24297123

[B9] Choi M. H. Jung J. I. Chung W. D. Kim Y. J. Lee S. E. Han D. H. . (2014). Acute pulmonary complications in patients with hematologic malignancies. Radiographics 34 (6), 1755–1768. doi: 10.1148/rg.346130107 25310429

[B10] Cole J. R. Wang Q. Fish J. A. Chai B. McGarrell D. M. Sun Y. . (2014). Ribosomal database project: data and tools for high throughput rRNA analysis. Nucleic Acids Res. 42 (Database issue), D633–D642. doi: 10.1093/nar/gkt1244 24288368 PMC3965039

[B11] Davis N. M. Proctor D. M. Holmes S. P. Relman D. A. Callahan B. J. (2018). Simple statistical identification and removal of contaminant sequences in marker-gene and metagenomics data. Microbiome 6 (1), 226. doi: 10.1186/s40168-018-0605-2 30558668 PMC6298009

[B12] DeSantis T. Z. Hugenholtz P. Larsen N. Rojas M. Brodie E. L. Keller K. . (2006). Greengenes, a chimera-checked 16S rRNA gene database and workbench compatible with ARB. Appl. Environ. Microbiol. 72 (7), 5069–5072. doi: 10.1128/AEM.03006-05 16820507 PMC1489311

[B13] Dickson R. P. Erb-Downward J. R. Freeman C. M. McCloskey L. Falkowski N. R. Huffnagle G. B. . (2017). Bacterial topography of the healthy human lower respiratory tract. mBio 8 (1), e02287–16. doi: 10.1128/mBio.02287-16 28196961 PMC5312084

[B14] Dickson R. P. Erb-Downward J. R. Martinez F. J. Huffnagle G. B. (2016a). The microbiome and the respiratory tract. Annu. Rev. Physiol. 78, 481–504. doi: 10.1146/annurev-physiol-021115-105238 26527186 PMC4751994

[B15] Dickson R. P. Martinez F. J. Huffnagle G. B. (2014). The role of the microbiome in exacerbations of chronic lung diseases. Lancet 384 (9944), 691–702. doi: 10.1016/S0140-6736(14)61136-3 25152271 PMC4166502

[B16] Dickson R. P. Schultz M. J. van der Poll T. Schouten L. R. Falkowski N. R. Luth J. E. . (2020). Lung microbiota predict clinical outcomes in critically ill patients. Am. J. Respir. Crit. Care Med. 201 (5), 555–563. doi: 10.1164/rccm.201907-1487OC 31973575 PMC7047465

[B17] Dickson R. P. Singer B. H. Newstead M. W. Falkowski N. R. Erb-Downward J. R. Standiford T. J. . (2016b). Enrichment of the lung microbiome with gut bacteria in sepsis and the acute respiratory distress syndrome. Nat. Microbiol. 1 (10), 16113. doi: 10.1038/nmicrobiol.2016.113 27670109 PMC5076472

[B18] Douglas G. M. Maffei V. J. Zaneveld J. R. Yurgel S. N. Brown J. R. Taylor C. M. . (2020). PICRUSt2 for prediction of metagenome functions. Nat. Biotechnol. 38 (6), 685–688. doi: 10.1038/s41587-020-0548-6 32483366 PMC7365738

[B19] Du B. Shen N. Tao Y. Sun S. Zhang F. Ren H. . (2021). Analysis of gut microbiota alteration and application as an auxiliary prognostic marker for sepsis in children: a pilot study. Transl. Pediatr. 10 (6), 1647–1657. doi: 10.21037/tp-21-51 34295779 PMC8261590

[B20] Edgar R. C. (2016). UNOISE2: Improved error-correction for illumina 16S and ITS amplicon sequencing. BioRxiv. Available at: https://www.biorxiv.org/content/10.1101/081257v1

[B21] Edgar R. C. Haas B. J. Clemente J. C. Quince C. Knight R. (2011). UCHIME improves sensitivity and speed of chimera detection. Bioinformatics 27 (16), 2194–2200. doi: 10.1093/bioinformatics/btr381 21700674 PMC3150044

[B22] Golob J. L. Pergam S. A. Srinivasan S. Fiedler T. L. Liu C. Garcia K. . (2017). Stool microbiota at neutrophil recovery is predictive for severe acute graft vs host disease after hematopoietic cell transplantation. Clin. Infect. Dis. 65 (12), 1984–1991. doi: 10.1093/cid/cix699 29020185 PMC5850019

[B23] Gooley T. A. Chien J. W. Pergam S. A. Hingorani S. Sorror M. L. Boeckh M. . (2010). Reduced mortality after allogeneic hematopoietic-cell transplantation. N Engl. J. Med. 363 (22), 2091–2101. doi: 10.1056/NEJMoa1004383 21105791 PMC3017343

[B24] Harris B. Morjaria S. M. Littmann E. R. Geyer A. I. Stover D. E. Barker J. N. . (2016). Gut microbiota predict pulmonary infiltrates after allogeneic hematopoietic cell transplantation. Am. J. Respir. Crit. Care Med. 194 (4), 450–463. doi: 10.1164/rccm.201507-1491OC 26886180 PMC5003327

[B25] Heidrich V. Bruno J. S. Knebel F. H. de Molla V. C. Miranda-Silva W. Asprino P. F. . (2021). Dental biofilm microbiota dysbiosis is associated with the risk of acute graft-Versus-Host disease after allogeneic hematopoietic stem cell transplantation. Front. Immunol. 12. doi: 10.3389/fimmu.2021.692225 PMC8250416 34220852

[B26] He Y. Yu W. Ning P. Luo Q. Zhao L. Xie Y. . (2022). Shared and specific lung microbiota with metabolic profiles in bronchoalveolar lavage fluid between infectious and inflammatory respiratory diseases. J. Inflammation Res. 15, 187–198. doi: 10.2147/JIR.S342462 PMC8760989 35046693

[B27] Huang Y. J. Charlson E. S. Collman R. G. Colombini-Hatch S. Martinez F. D. Senior R. M. (2013). The role of the lung microbiome in health and disease. a national heart, lung, and blood institute workshop report. Am. J. Respir. Crit. Care Med. 187 (12), 1382–1387. doi: 10.1164/rccm.201303-0488WS 23614695 PMC5155250

[B28] Hubbell S. P. (2010). Neutral Theory and the Theory of Island Biogeography. Princeton: Princeton University Press.

[B29] Huffnagle G. B. Dickson R. P. Lukacs N. W. (2017). The respiratory tract microbiome and lung inflammation: A two-way street. Mucosal Immunol. 10 (2), 299–306. doi: 10.1038/mi.2016.108 27966551 PMC5765541

[B30] Invernizzi R. Lloyd C. M. Molyneaux P. L. (2020). Respiratory microbiome and epithelial interactions shape immunity in the lungs. Immunology 160 (2), 171–182. doi: 10.1111/imm.13195 32196653 PMC7218407

[B31] Jari Oksanen F. G. B. Frienly M. Kindt R. Legendre P. McGlinn D. R.Minchin P. . (2020) Vegan: Community ecology package. Available at: https://github.com/vegandevs/vegan.

[B32] Jorth P. Ehsan Z. Rezayat A. Caldwell E. Pope C. Brewington J. J. . (2019). Direct lung sampling indicates that established pathogens dominate early infections in children with cystic fibrosis. Cell Rep. 27 (4), 1190–1204.e1193. doi: 10.1016/j.celrep.2019.03.086 31018133 PMC6668708

[B33] Kader H. A. Khanna S. Hutchinson R. M. Aukett R. J. Archer J. (1994). Pulmonary complications of bone marrow transplantation: the impact of variations in total body irradiation parameters. Clin. Oncol. (R Coll. Radiol) 6 (2), 96–101. doi: 10.1016/s0936-6555(05)80111-6 8018580

[B34] Kalil A. C. Metersky M. L. Klompas M. Muscedere J. Sweeney D. A. Palmer L. B. . (2016). Management of adults with hospital-acquired and ventilator-associated pneumonia: 2016 clinical practice guidelines by the infectious diseases society of America and the American thoracic society. Clin. Infect. Dis. 63 (5), e61–e111. doi: 10.1093/cid/ciw353 27418577 PMC4981759

[B35] Kanehisa M. Furumichi M. Sato Y. Ishiguro-Watanabe M. Tanabe M. (2021). KEGG: integrating viruses and cellular organisms. Nucleic Acids Res. 49 (D1), D545–D551. doi: 10.1093/nar/gkaa970 33125081 PMC7779016

[B36] Karstens L. Asquith M. Davin S. Fair D. Gregory W. T. Wolfe A. J. . (2019). Controlling for contaminants in low-biomass 16S rRNA gene sequencing experiments. mSystems 4 (4), e00290-19. doi: 10.1128/mSystems.00290-19 PMC6550369 31164452

[B37] Kembel S. W. Cowan P. D. Helmus M. R. Cornwell W. K. Morlon H. Ackerly D. D. . (2010). Picante: R tools for integrating phylogenies and ecology. Bioinformatics 26 (11), 1463–1464. doi: 10.1093/bioinformatics/btq166 20395285

[B38] Klindworth A. Pruesse E. Schweer T. Peplies J. Quast C. Horn M. . (2013). Evaluation of general 16S ribosomal RNA gene PCR primers for classical and next-generation sequencing-based diversity studies. Nucleic Acids Res. 41 (1), e1. doi: 10.1093/nar/gks808 22933715 PMC3592464

[B39] Kotloff R. M. Ahya V. N. Crawford S. W. (2004). Pulmonary complications of solid organ and hematopoietic stem cell transplantation. Am. J. Respir. Crit. Care Med. 170 (1), 22–48. doi: 10.1164/rccm.200309-1322SO 15070821

[B40] Kurtz Z. D. Muller C. L. Miraldi E. R. Littman D. R. Blaser M. J. Bonneau R. A. (2015). Sparse and compositionally robust inference of microbial ecological networks. PloS Comput. Biol. 11 (5), e1004226. doi: 10.1371/journal.pcbi.1004226 25950956 PMC4423992

[B41] Lonergan Z. R. Palmer L. D. Skaar E. P. (2020). Histidine utilization is a critical determinant of acinetobacter pathogenesis. Infect. Immun. 88 (7), e00118–20. doi: 10.1128/IAI.00118-20 32341119 PMC7309604

[B42] Lucena C. M. Torres A. Rovira M. Marcos M. A. de la Bellacasa J. P. Sanchez M. . (2014). Pulmonary complications in hematopoietic SCT: A prospective study. Bone Marrow Transplant. 49 (10), 1293–1299. doi: 10.1038/bmt.2014.151 25046219 PMC7094728

[B43] Mandell L. A. Wunderink R. G. Anzueto A. Bartlett J. G. Campbell G. D. Dean N. C. . (2007). Infectious diseases society of America/American thoracic society consensus guidelines on the management of community-acquired pneumonia in adults. Clin. Infect. Dis. 44 Suppl 2, S27–S72. doi: 10.1086/511159 17278083 PMC7107997

[B44] Marsland B. J. Gollwitzer E. S. (2014). Host-microorganism interactions in lung diseases. Nat. Rev. Immunol. 14 (12), 827–835. doi: 10.1038/nri3769 25421702

[B45] Martin-Loeches I. Dickson R. Torres A. Hanberger H. Lipman J. Antonelli M. . (2020). The importance of airway and lung microbiome in the critically ill. Crit. Care 24 (1), 537. doi: 10.1186/s13054-020-03219-4 32867808 PMC7457224

[B46] Meehan C. J. Barco R. A. Loh Y. E. Cogneau S. Rigouts L. (2021). Reconstituting the genus mycobacterium. Int. J. Syst. Evol. Microbiol. 71 (9), 004922. doi: 10.1099/ijsem.0.004922 PMC8549266 34554081

[B47] Nearing J. T. Douglas G. M. Hayes M. G. MacDonald J. Desai D. K. Allward N. . (2022). Microbiome differential abundance methods produce different results across 38 datasets. Nat. Commun. 13 (1), 342. doi: 10.1038/s41467-022-28034-z 35039521 PMC8763921

[B48] Nusair S. Breuer R. Shapira M. Y. Berkman N. Or R. (2004). Low incidence of pulmonary complications following nonmyeloablative stem cell transplantation. Eur. Respir. J. 23 (3), 440–445. doi: 10.1183/09031936.04.00053004 15065836

[B49] O’Dwyer D. N. Dickson R. P. Moore B. B. (2016). The lung microbiome, immunity, and the pathogenesis of chronic lung disease. J. Immunol. 196 (12), 4839–4847. doi: 10.4049/jimmunol.1600279 27260767 PMC4894335

[B50] O’Dwyer D. N. Zhou X. Wilke C. A. Xia M. Falkowski N. R. Norman K. C. . (2018). Lung dysbiosis, inflammation, and injury in hematopoietic cell transplantation. Am. J. Respir. Crit. Care Med. 198 (10), 1312–1321. doi: 10.1164/rccm.201712-2456OC 29878854 PMC6290939

[B51] Pattaroni C. Watzenboeck M. L. Schneidegger S. Kieser S. Wong N. C. Bernasconi E. . (2018). Early-life formation of the microbial and immunological environment of the human airways. Cell Host Microbe 24 (6), 857–865.e854. doi: 10.1016/j.chom.2018.10.019 30503510

[B52] Peters S. G. Afessa B. (2005). Acute lung injury after hematopoietic stem cell transplantation. Clin. Chest Med. 26 (4), 561–569, vi. doi: 10.1016/j.ccm.2005.06.009 16263396

[B53] Robinson M. D. McCarthy D. J. Smyth G. K. (2010). edgeR: a bioconductor package for differential expression analysis of digital gene expression data. Bioinformatics 26 (1), 139–140. doi: 10.1093/bioinformatics/btp616 19910308 PMC2796818

[B54] Rognes T. Flouri T. Nichols B. Quince C. Mahe F. (2016). VSEARCH: a versatile open source tool for metagenomics. PeerJ 4, e2584. doi: 10.7717/peerj.2584 27781170 PMC5075697

[B55] Salter S. J. Cox M. J. Turek E. M. Calus S. T. Cookson W. O. Moffatt M. F. . (2014). Reagent and laboratory contamination can critically impact sequence-based microbiome analyses. BMC Biol. 12, 87. doi: 10.1186/s12915-014-0087-z 25387460 PMC4228153

[B56] Segata N. Izard J. Waldron L. Gevers D. Miropolsky L. Garrett W. S. . (2011). Metagenomic biomarker discovery and explanation. Genome Biol. 12 (6), R60. doi: 10.1186/gb-2011-12-6-r60 21702898 PMC3218848

[B57] Shono Y. Docampo M. D. Peled J. U. Perobelli S. M. Velardi E. Tsai J. J. . (2016). Increased GVHD-related mortality with broad-spectrum antibiotic use after allogeneic hematopoietic stem cell transplantation in human patients and mice. Sci. Transl. Med. 8 (339), 339ra371. doi: 10.1126/scitranslmed.aaf2311 PMC4991773 27194729

[B58] Singanayagam A. Glanville N. Cuthbertson L. Bartlett N. W. Finney L. J. Turek E. . (2019). Inhaled corticosteroid suppression of cathelicidin drives dysbiosis and bacterial infection in chronic obstructive pulmonary disease. Sci. Transl. Med. 11 (507), eaav3879. doi: 10.1126/scitranslmed.aav3879 31462509 PMC7237237

[B59] Taur Y. Jenq R. R. Perales M. A. Littmann E. R. Morjaria S. Ling L. . (2014). The effects of intestinal tract bacterial diversity on mortality following allogeneic hematopoietic stem cell transplantation. Blood 124 (7), 1174–1182. doi: 10.1182/blood-2014-02-554725 24939656 PMC4133489

[B60] Venkataraman A. Bassis C. M. Beck J. M. Young V. B. Curtis J. L. Huffnagle G. B. . (2015). Application of a neutral community model to assess structuring of the human lung microbiome. mBio 6 (1), e02284–14. doi: 10.1128/mBio.02284-14 25604788 PMC4324308

[B61] Ward T. Larson J. Meulemans J. Hillmann B. Lynch J. Sidiropoulus D . (2017). BugBase predicts organism-level microbiome phenotypes. BioRxiv. Available at: https://www.biorxiv.org/content/10.1101/133462v1.

[B62] Zheng Y. Ning P. Luo Q. He Y. Yu X. Liu X. . (2019). Inflammatory responses relate to distinct bronchoalveolar lavage lipidome in community-acquired pneumonia patients: A pilot study. Respir. Res. 20 (1), 82. doi: 10.1186/s12931-019-1028-8 31046764 PMC6498485

[B63] Zhou X. O’Dwyer D. N. Xia M. Miller H. K. Chan P. R. Trulik K. . (2019). First-onset herpesviral infection and lung injury in allogeneic hematopoietic cell transplantation. Am. J. Respir. Crit. Care Med. 200 (1), 63–74. doi: 10.1164/rccm.201809-1635OC 30742492 PMC6603051

[B64] Zinter M. S. Hume J. R. (2021). Effects of hematopoietic cell transplantation on the pulmonary immune response to infection. Front. Pediatr. 9. doi: 10.3389/fped.2021.634566 PMC7871005 33575235

