# Supplementary material for: Characteristics of lower respiratory tract microbiota in the patients with post-hematopoietic stem cell transplantation pneumonia
Source: Front Cell Infect Microbiol. 2022 Sep 13;12:943317. doi: 10.3389/fcimb.2022.943317 (PMC9513191; doi:10.3389/fcimb.2022.943317)
Supplement: Supplementary file 1 [file DataSheet_1.docx]

**Supplementary materials**

**Contents of Supplements**

[Supplement 1 Supplementary Methods 3](#_Toc107850564)

[Supplement 2 Supplementary Tables 3](#_Toc107850565)

[Supplement 2 Supplementary Figures 7](#_Toc107850566)

# Supplement 1 Supplementary Methods

DNA concentration and purity were measured using agarose gels (1%). Then DNA was diluted to 1 ng µl‐1 using sterile water and used to amplify the V3-V4 region of 16S rRNA through polymerase chain reaction (PCR) with primers (338F and 806R) which were tagged with specific barcodes. All PCR reactions were carried out in 30 µl reactions, containing 15 µl of Phusion® High‐Fidelity PCR Master Mix with GC Buffer (New England Biolabs, USA), 10 ng template DNA and 0.2 µM of each primer. Thermal cycling began with an initial denaturation for 1 min at 98°C, followed by 30 cycles of denaturation for 10 s at 98°C, annealing for 30 s at 50°C and elongation for 30 s at 72°C, followed by extension for 5 min at 72°C.The PCR products were detected by agarose gel electrophoresis (2%) and then mixed in equidensity ratios. The mixture of PCR products was purified with the QIAEX II Gel Extraction Kit (QIAGEN, Germany). Sequencing libraries were generated using TruSeq® DNA PCR-Free Sample Preparation Kit (Illumina, USA) following the manufacturer's instructions. The quality of libraries was determined using the Qubit Fluorometer (Thermo Scientific, USA). The high‐quality libraries were sequenced on the HiSeq2500 platform and 250 bp paired-end raw reads were generated at Novogene (Tianjin, China).

# Supplement 2 Supplementary Tables

e-Table 1. Demographic and baseline characteristics of patients with post-HSCT pneumonia.

|  | HSCT patients with PCs (n=55) |
| --- | --- |
| Age at HSCT, years | 34 (27-43) |
| Gender, n, (% male) | 40 (72.7%) |
| Time from diagnosis to transplant, days | 228 (167-442) |
| Phase of the onset of pneumonia after transplant | 165 (88.5-268) |
| pre-engraftment (neutropenic) phase | 4 (7.3%) |
| early post-engraftment phase | 16 (29.1%) |
| late post-engraftment phase | 35 (63.6%) |
| Time from transplant to the onset of pneumonia, days | 165 (88.5-260) |
| Indication for HSCT, no. (%) |  |
| ALL | 16 (29.1%) |
| AML | 26 (47.3%) |
| CML | 3 (5.5%) |
| CLL | 1 (1.8%) |
| MDS | 7 (12.7%) |
| Lymphoma | 2 (3.6%) |
| HLA disparity, no. (%) |  |
| Matched | 38 (69.1%) |
| Mismatched | 17 (30.9%) |
| Donor relationship, no. (%) |  |
| Related | 51 (92.7%) |
| Unrelated | 4 (7.3%) |
| Presence of GVHD, no. (%) |  |
| Acute GVHD | 25 (45.5%) |
| Chronic GVHD | 7 (12.7%) |

e-Table 2. Demographic and baseline characteristics of non-survivors and survivors in patients with post-HSCT pneumonia

|  | Non-Survivor  (n = 18) | Survivor  (n = 37) | *p*-value |
| --- | --- | --- | --- |
| Age | 33(28-37.5) | 36(27-46) | 0.294 |
| Gender, n, (% male) | 16 (88.9) | 24(64.9) | 0.105 |
| GVHD, n, (%) | 13 | 19 | 0.141 |
| Laboratory Findings |  |  |  |
| Peripheral blood |  |  |  |
| WBC (× 10^9/^L) | 4.50(2.07-7.40) | 4.10(2.80-6.16) | 0.900 |
| Neutrophils (%) | 79.85(59.58-88.38) | 73.10(59.75-84.65) | 0.473 |
| Lymphocytes (%) | 11.70(6.08-28.15) | 18.20(8.95-24.40) | 0.404 |
| BAL related |  |  |  |
| PMN percentages (%) | 22.75(4.13-52.5) | 7.00(1.00-17.50) | 0.018 |
| Lymphocyte percentages (%) | 26.50(9.50-43.75) | 42.00 (17.00-54.50) | 0.139 |
| Eosinophil percentages (%) | 0.00(0.00-0.25) | 0.00(0.00-1.00) | 0.897 |
| Macrophages percentages (%) | 35.5(15.50-54.50) | 42.00(23.5-67.50) | 0.462 |
| Inflammatory markers |  |  |  |
| PCT (μg/L) | 0.50(0.25-1.67) | 0.13(0.10-0.25) | 0.002 |
| CRP (mg/L) | 55.62(8.59-116.17) | 17.23(3.60-76.57) | 0.103 |
| Ventilation, no. (%) |  |  |  |
| HFNC or noninvasive ventilation | 0 | 3(8.1) | <0.000 |
| Intubation and invasive ventilation | 10(58.8) | 0 | <0.000 |

e-Table 3. Symptoms, causative pathogens, therapies, and outcomes of patients with post-HSCT pneumonia and CAP patients. Recent drug usage means in the last three months.

|  | HSCT patients with PCs (n=55) | CAP (N = 44) | *P*-value |
| --- | --- | --- | --- |
| Symptoms |  |  |  |
| Fever, no. (%) | 34(61.8) | 39(88.6) | 0.475 |
| Cough, no. (%) | 18(32.7) | 31(70.5) | <0.001 |
| Chest tightness, no. (%) | 18(32.7) | 10(22.7) | 0.272 |
| Respiratory failure, no. (%) | 10(18.2) | 16(36.4) | 0.041 |
| Detected pathogen, no. (%) |  |  |  |
| Bacteria |  |  |  |
| *Staphylococcus aureus* | 0(0%) | 3(6.8%) | 0.169 |
| *Acinetobacter baumannii* | 0(0%) | 1(2.3%) | 0.201 |
| *Klebsiella pneumoniae* | 2(3.6%) | 2(4.5%) | 0.819 |
| *Pseudomonas aeruginosa* | 2(3.6%) | 1(2.3%) | 0.694 |
| *Escherichia coli* | 1(1.8%) | 0(0%) | 0.369 |
| *Mycobacterium tuberculosis* | 3(5.5%) | 0(0%) | 0.325 |
| Virus | 13 (23.6%) | 12(27.3%) | 0.379 |
| Fungus | 12 (21.8%) | 3(6.8%) | 0.039 |
| Atypical pathogen | 0(0%) | 7(15.9%) | 0.007 |
| Medication history, no. (%) |  |  |  |
| Recent corticosteroid usage | 34 (61.8%) | 0(0%) | <0.001 |
| Recent antibiotic usage | 49 (89.1%) | 37(84.1%) | 0.318 |
| Recent antifungal usage | 45 (81.8%) | 1(2.3%) | <0.001 |
| Recent antiviral usage | 35 (63.6%) | 21(47.7%) | 0.113 |
| *Pneumocystis carinii* pneumonia prophylaxis | 30(54.5) | 0(0%) | <0.001 |
| Ventilation, no. (%) |  |  |  |
| HFNC or noninvasive ventilation | 3 (5.4%) | 6(13.6%) | 0.291 |
| Intubation and invasive ventilation, n (%) | 10 (18.2%) | 7 (18.2%) | - |
| Outcome, no. (%) |  |  |  |
| Death | 18 (32.7%) | 6 (13.6%) | 0.028 |

e-Table 5. The occurrence and average relative abundance of contamination genera detected in the negative sequencing blank controls compared to Salter et al. The red font referred to genera that have not been detected in pulmonary specimens in previous studies

| Genus | Prevalence (rel abundance > 0) | Prevalence (rel abundance > 0.1) | Average relative abundance |
| --- | --- | --- | --- |
| Alphaproteobacteria |  |  |  |
| *Afipia* | 0 | 0 | 0 |
| *Aquabacterium* | 0 | 0 | 0 |
| *Asticcacaulis* | 0 | 0 | 0 |
| *Aurantimonas* | 0 | 0 | 0 |
| *Beijerinckia* | 0 | 0 | 0 |
| *Bosea* | 0 | 0 | 0 |
| *Bradyhizobium* | 0 | 0 | 0 |
| *Brevundimonas* | 0 | 0 | 0 |
| *Caulobacter* | 0 | 0 | 0 |
| *Craurococcus* | 0 | 0 | 0 |
| *Devosia* | 0 | 0 | 0 |
| *Hoeflea* | 0 | 0 | 0 |
| *Mesorhizobium* | 0 | 0 | 0 |
| *Methylobacterium* | 0 | 0 | 0 |
| *Novosphingobioum* | 0 | 0 | 0 |
| *Ochrobactrum* | 0.17 | 0 | 0.0000615247 |
| *Paracoccus* | 0 | 0 | 0 |
| *Pedomicrobiom* | 0 | 0 | 0 |
| *Phyllobacterium* | 0 | 0 | 0 |
| *Rhizobium* | 0 | 0 | 0 |
| *Roseomonas* | 0 | 0 | 0 |
| *Sphingobium* | 0.61 | 0.007 | 0.0041589 |
| *Sphingomonas* | 0.97 | 0.07 | 0.03127 |
| *Sphingopyxis* | 0.29 | 0.007 | 0.0001512 |
| *Betaproteobacteria* |  |  |  |
| *Acidovorax* | 0.76 | 0.007 | 0.005724 |
| *Azoarcus* | 0 | 0 | 0 |
| *Azospira* | 0 | 0 | 0 |
| *Burkholderia* | 0.19 | 0.015 | 0.00362 |
| *Comamonas* | 0.18 | 0 | 0.000120534 |
| *Cupriavidus* | 0.46 | 0 | 0.00018774 |
| *Curvibacter* | 0.14 | 0 | 7.0513E-05 |
| *Delftia* | 0.54 | 0.007 | 0.0019020 |
| *Duganella* | 0 | 0 | 0 |
| *Herbaspirillum* | 0 | 0 | 0 |
| *Janthinobacterium* | 0 | 0 | 0 |
| *Kingella* | 0 | 0 | 0 |
| *Leptothrix* | 0 | 0 | 0 |
| *Limnobacter* | 0.24 | 0 | 0.000111891 |
| *Massilia* | 0 | 0 | 0 |
| *Methylophilus* | 0.07 | 0 | 2.2533E-05 |
| *Methyloversatilis* | 0 | 0 | 0 |
| *Oxalobacter* | 0 | 0 | 0 |
| *Pelomonas* | 0.26 | 0.007 | 0.001528 |
| *Polaromonas* | 0.4 | 0 | 0.000142261 |
| *Ralstonia* | 0.5 | 0 | 0.00033014 |
| *Schlegelella* | 0 | 0 | 0 |
| *Sulfuritalea* | 0 | 0 | 0 |
| *Undibacterium* | 0.09 | 0 | 2.73589E-05 |
| *Variovorax* | 0 | 0 | 0 |
| Gammaproteobacteria |  |  |  |
| *Acinetobacter* | 0.99 | 0.015 | 0.022232401 |
| *Enhydrobacter* | 0 | 0 | 0 |
| *Enterobacter* | 0 | 0 | 0 |
| *Escherichia* | 0 | 0 | 0 |
| *Nevskia* | 0.28 | 0 | 0.000412552 |
| *Pseudomonas* | 0.96 | 0.05 | 0.02625 |
| *Pseudoxanthomonas* | 0.25 | 0.007 | 0.001376 |
| *Psychobacter* | 0 | 0 | 0 |
| *Stenotrophomonas* | 0.84 | 0.007 | 0.003077529 |
| *Xanthomonas* | 0 | 0 | 0 |
| Actinobacteria |  |  |  |
| *Aeromonas* | 0.39 | 0.007 | 0.002979 |
| *Arthrobacter* | 0 | 0 | 0 |
| *Beutenbergia* | 0 | 0 | 0 |
| *Brevibacterium* | 0.33 | 0 | 9.67592E-05 |
| *Corynebacterium* | 0.94 | 0 | 0.002884621 |
| *Curtobacterium* | 0 | 0 | 0 |
| *Dietzia* | 0.62 | 0 | 0.00035435 |
| *Geodermatophilus* | 0 | 0 | 0 |
| *Janibacter* | 0 | 0 | 0 |
| *Kocuria* | 0 | 0 | 0 |
| *Microbacterium* | 0 | 0 | 0 |
| *Micrococcus* | 0 | 0 | 0 |
| *Microlunatus* | 0 | 0 | 0 |
| *Patulibacter* | 0 | 0 | 0 |
| *Propionibacterum* | 0 | 0 | 0 |
| *Rhodococcus* | 0 | 0 | 0 |
| *Tsukamurella* | 0 | 0 | 0 |
| Firmicutes |  |  |  |
| *Abiotrophia* | 0 | 0 | 0 |
| *Bacillus* | 0.96 | 0.06 | 0.03075925 |
| *Brevibacillus* | 0 | 0 | 0 |
| *Brochothrix* | 0 | 0 | 0 |
| *Facklamia* | 0.27 | 0 | 0.000119166 |
| *Paenibacillus* | 0 | 0 | 0 |
| *Streptococcus* | 0.92 | 0.007 | 0.00816924 |
| Bacteroidetes |  |  |  |
| *Chryseobacterium* | 0 | 0 | 0 |
| *Dyadobacter* | 0.25 | 0.007 | 0.0001087873 |
| *Flavobacterium* | 0 | 0 | 0 |
| *Hydrotalea* | 0 | 0 | 0 |
| *Niatella* | 0 | 0 | 0 |
| *Olivibacter* | 0 | 0 | 0 |
| *Pedobacter* | 0.23 | 0.015 | 0.000265886 |
| *Wautersiella* | 0 | 0 | 0 |
| Deinococcus-Thermus |  |  |  |
| *Deinococcus* | 0.42 | 0 | 0.000141132 |

e-Table 6. The read counts in per samples and the range, mean and standard deviation (SD) of read counts in each group.

| Sample | Reads |  | Sample | Reads |  | Sample | Reads |
| --- | --- | --- | --- | --- | --- | --- | --- |
| sam 1 | 30159 |  | sam 44 | 30317 |  | sam 87 | 29704 |
| sam 2 | 30254 |  | sam 45 | 29952 |  | sam 88 | 30092 |
| sam 3 | 30266 |  | sam 46 | 29823 |  | sam 89 | 30098 |
| sam 4 | 30318 |  | sam 47 | 30185 |  | sam 90 | 29787 |
| sam 5 | 30172 |  | sam 48 | 30216 |  | sam 91 | 30136 |
| sam 6 | 30228 |  | sam 49 | 29691 |  | sam 92 | 30244 |
| sam 7 | 29828 |  | sam 50 | 29752 |  | sam 93 | 30072 |
| sam 8 | 30343 |  | sam 51 | 29830 |  | sam 94 | 30109 |
| sam 9 | 30091 |  | sam 52 | 29832 |  | sam 95 | 30097 |
| sam 10 | 29729 |  | sam 53 | 29890 |  | sam 96 | 30207 |
| sam 11 | 30690 |  | sam 54 | 29802 |  | sam 97 | 30199 |
| sam 12 | 30065 |  | sam 55 | 29685 |  | sam 98 | 29431 |
| sam 13 | 29534 |  | sam 56 | 30244 |  | sam 99 | 30613 |
| sam 14 | 30320 |  | sam 57 | 30247 |  | sam 100 | 30240 |
| sam 15 | 29899 |  | sam 58 | 30243 |  | sam 101 | 30243 |
| sam 16 | 30179 |  | sam 59 | 29577 |  | sam 102 | 30208 |
| sam 17 | 29832 |  | sam 60 | 30224 |  | sam 103 | 30219 |
| sam 18 | 30189 |  | sam 61 | 30257 |  | sam 104 | 30245 |
| sam 19 | 30346 |  | sam 62 | 30224 |  | sam 105 | 30186 |
| sam 20 | 30207 |  | sam 63 | 30278 |  | sam 106 | 30155 |
| sam 21 | 30259 |  | sam 64 | 29869 |  | sam 107 | 30273 |
| sam 22 | 30570 |  | sam 65 | 30079 |  | sam 108 | 30170 |
| sam 23 | 29731 |  | sam 66 | 29647 |  | sam 109 | 30400 |
| sam 24 | 30496 |  | sam 67 | 29866 |  | sam 110 | 30219 |
| sam 25 | 30376 |  | sam 68 | 30183 |  | sam 111 | 30501 |
| sam 26 | 29976 |  | sam 69 | 30349 |  | sam 112 | 30159 |
| sam 27 | 30375 |  | sam 70 | 30124 |  | sam 113 | 30091 |
| sam 28 | 30076 |  | sam 71 | 30152 |  | sam 114 | 30258 |
| sam 29 | 30366 |  | sam 72 | 30269 |  | sam 115 | 30068 |
| sam 30 | 30144 |  | sam 73 | 30255 |  | sam 116 | 30349 |
| sam 31 | 30371 |  | sam 74 | 30138 |  | sam 117 | 30081 |
| sam 32 | 30443 |  | sam 75 | 29583 |  | sam 118 | 29750 |
| sam 33 | 29693 |  | sam 76 | 30108 |  | sam 119 | 30171 |
| sam 34 | 30301 |  | sam 77 | 29906 |  | sam 120 | 30265 |
| sam 35 | 29646 |  | sam 78 | 30163 |  | sam 121 | 30245 |
| sam 36 | 29884 |  | sam 79 | 29906 |  | sam 122 | 30184 |
| sam 37 | 30182 |  | sam 80 | 30469 |  | sam 123 | 29775 |
| sam 38 | 30219 |  | sam 81 | 29808 |  | sam 124 | 30107 |
| sam 39 | 30297 |  | sam 82 | 29726 |  | sam 125 | 30123 |
| sam 40 | 29729 |  | sam 83 | 30284 |  | sam 126 | 30205 |
| sam 41 | 30255 |  | sam 84 | 30476 |  | sam 127 | 30178 |
| sam 42 | 29705 |  | sam 85 | 29540 |  | sam 128 | 30461 |
| sam 43 | 30122 |  | sam 86 | 30198 |  | sam 129 | 30160 |

| Group | HSCT_P | CAP | HC |
| --- | --- | --- | --- |
| Range | 29431-30613 | 29534-30690 | 29577-30443 |
| Mean | 30112.7 | 30160.8 | 30009.2 |
| SD | 241.6301 | 238.1616609 | 264.1527358 |

e-Table 4. Potential contaminations detected in the negative controls.

e-Table 7. The differential genera between the post-HSCT pneumonia and HC group base on the edgeR R package.

e-Table 8. The differential genera between the post-HSCT pneumonia and CAP group base on the edgeR R package.

e-Table 9. Differentially expressed pathways in the post-HSCT pneumonia group compared with HC group through a Wilcoxon Rank Sum Test.

e-Table 10. Differentially expressed pathways in the post-HSCT pneumonia group compared with CAP group through a Wilcoxon Rank Sum Test.

e-Table 11. The differential genera between the survivors and non-survivors in the post-HSCT pneumonia group base on the edgeR R package.

e-Table 12. The correlations between genera and clinical indicators.

# Supplement 2 Supplementary Figures


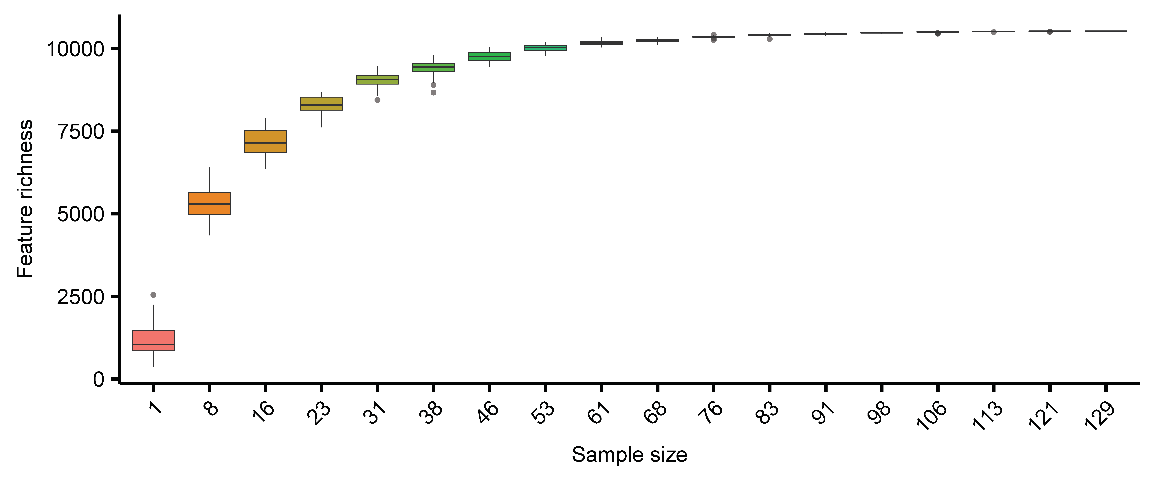


e-Figure 1. Species accumulation curves revealed that the sample size was sufficient to describe associated microbial community.


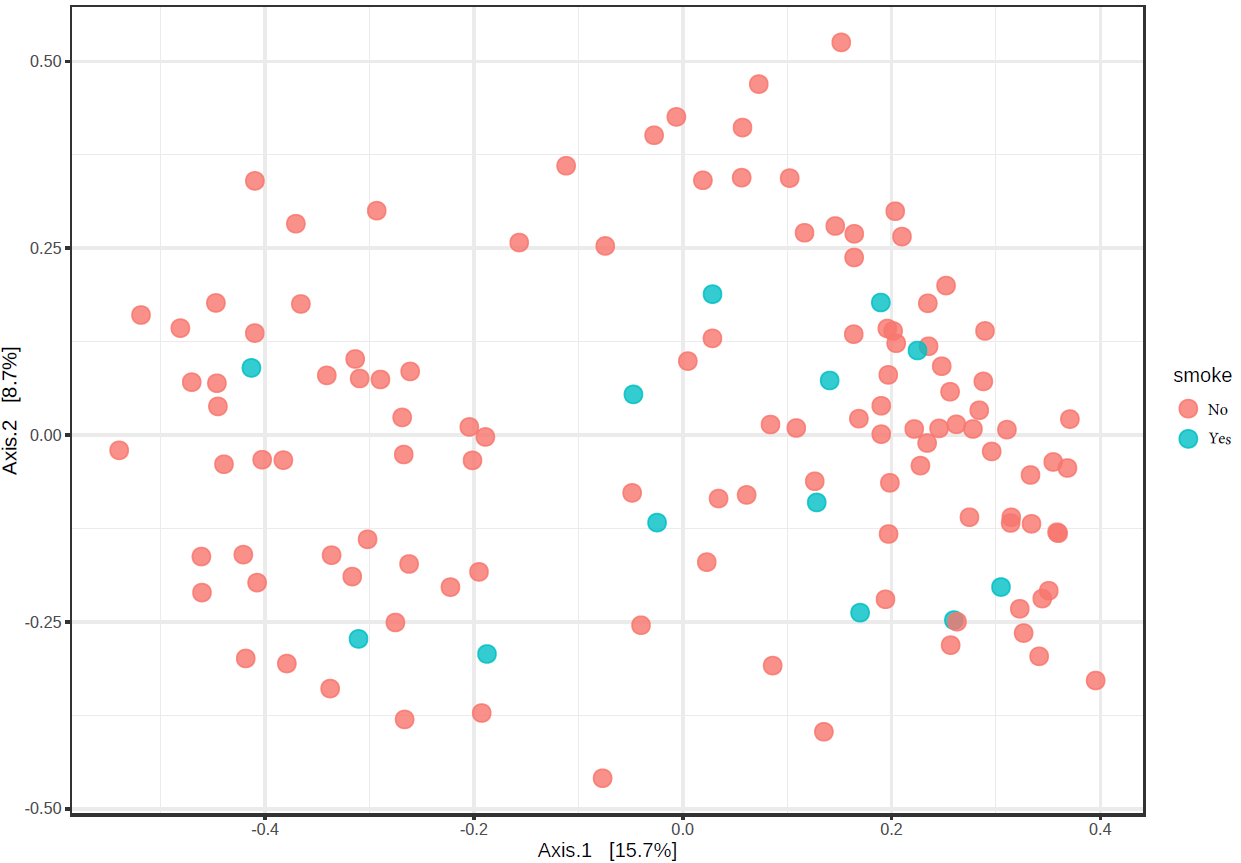


e-Figure 2A. Beta diversity was assessed by PERMANOVA based on Bray-Curtis distances using principal coordinate analysis (PCoA) between smoking and non-smoking subjects. There is no difference between smoking and non-smoking groups (*p* = 0.297).


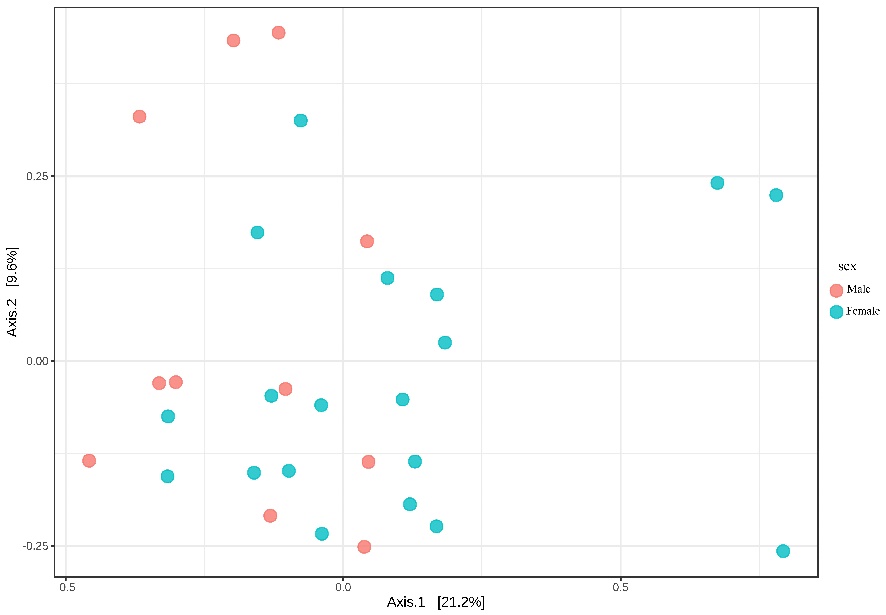


e-Figure 2B. Beta diversity was assessed by PERMANOVA based on Bray-Curtis distances using principal coordinate analysis (PCoA) between male and female in the healthy controls. There is no difference between the female or male (p = 0.19).


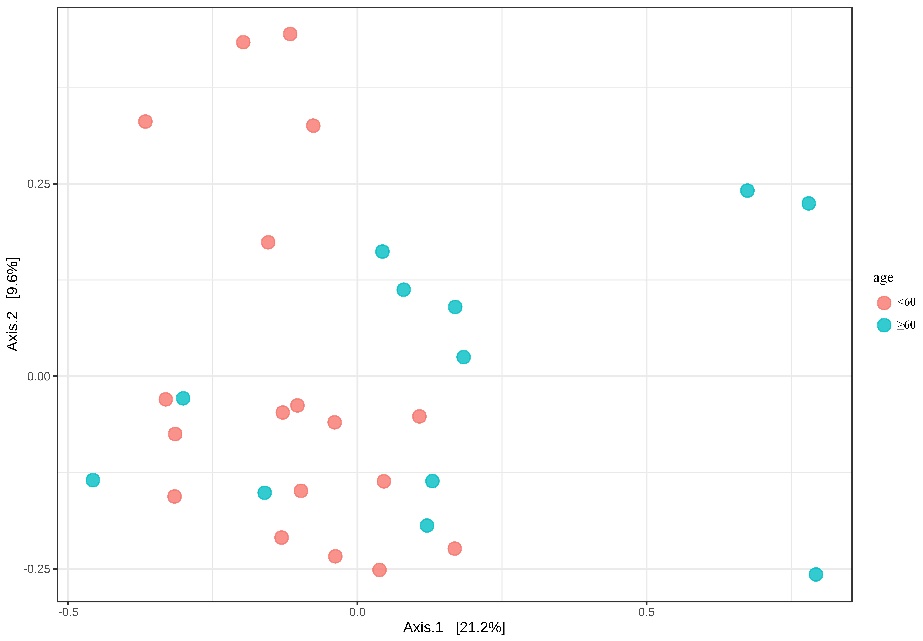


e-Figure 2C. Beta diversity was assessed by PERMANOVA based on Bray-Curtis distances using principal coordinate analysis (PCoA) between different age groups in the healthy controls. There is no difference between the two groups (p = 0.052).


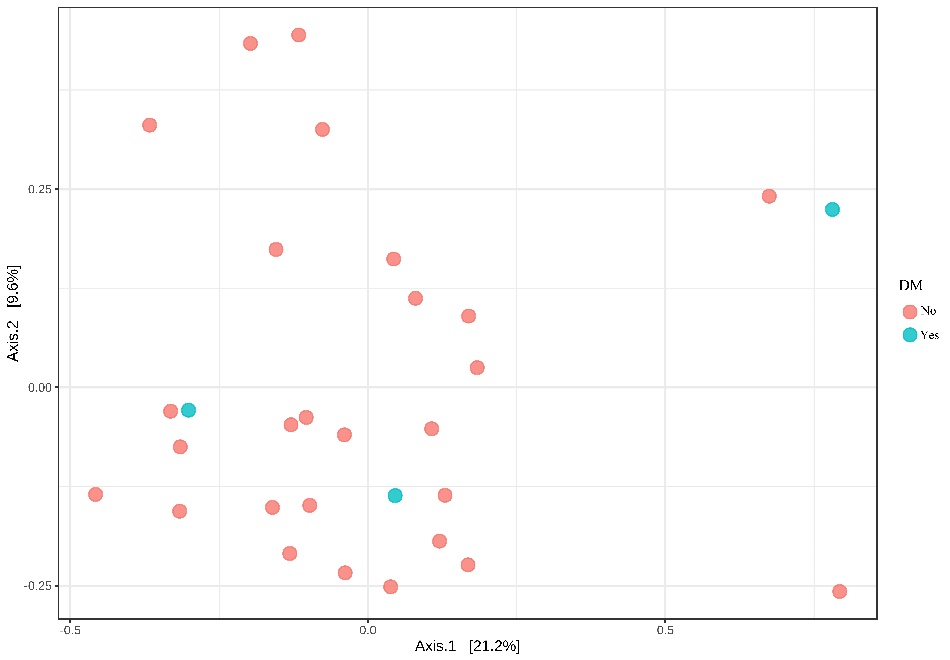


e-Figure 2D. Beta diversity was assessed by PERMANOVA based on Bray-Curtis distances using principal coordinate analysis (PCoA) between patients with or without diabetes mellitus in the healthy controls. There is no difference between the female or male (p = 0.223). DM, diabetes mellitus.


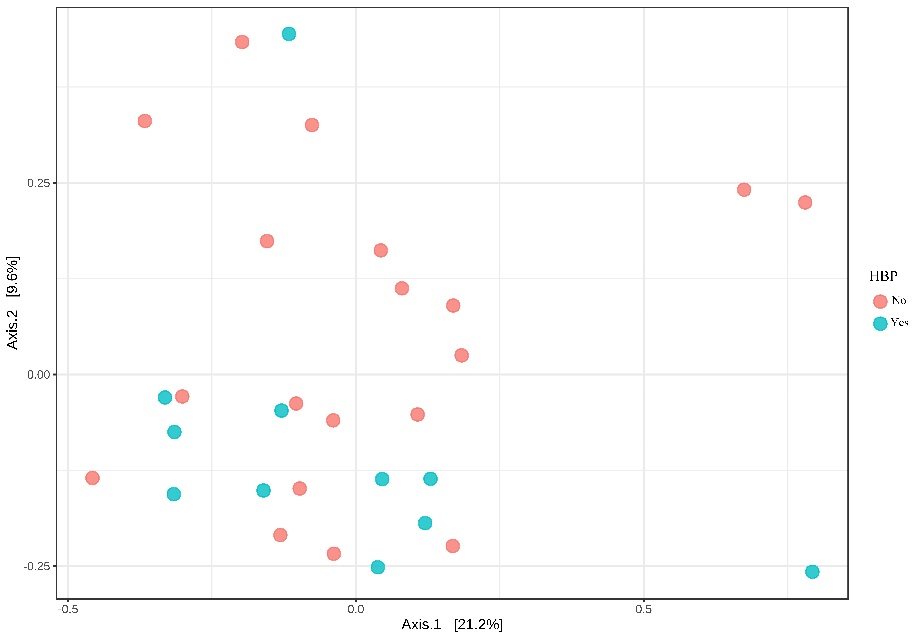


e-Figure 2E. Beta diversity was assessed by PERMANOVA based on Bray-Curtis distances using principal coordinate analysis (PCoA) between patients with or without hypertension in the healthy controls. There is no difference between the female or male (p = 0.922). HBP, hypertension.


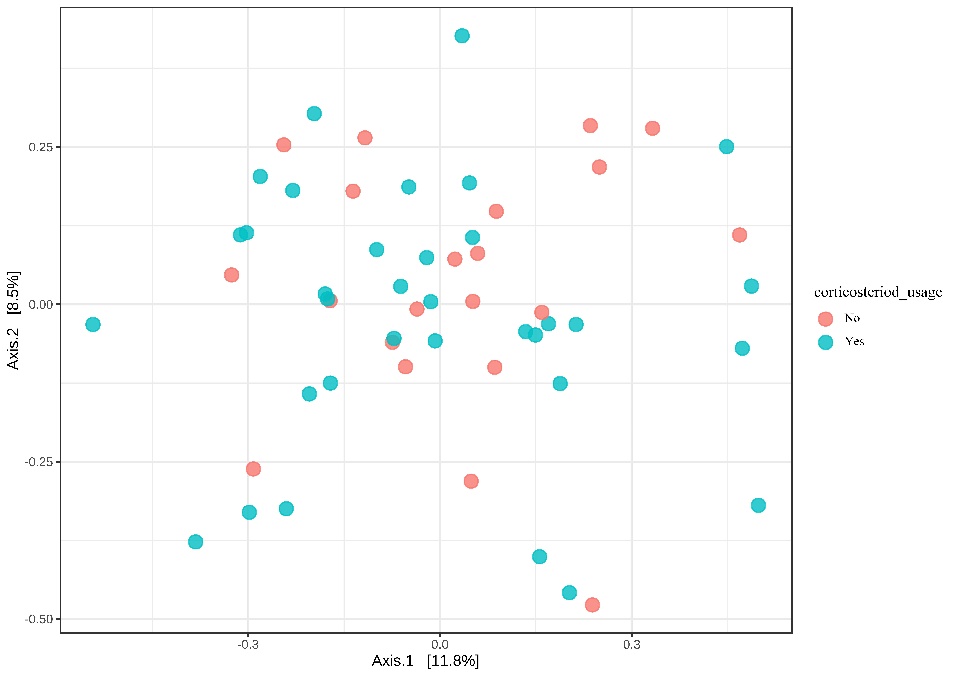


e-Figure 2F. Beta diversity was assessed by PERMANOVA based on Bray-Curtis distances using principal coordinate analysis (PCoA) between patients using corticosteroid or not using corticosteroid. There is no difference between these two groups (p = 0.552).


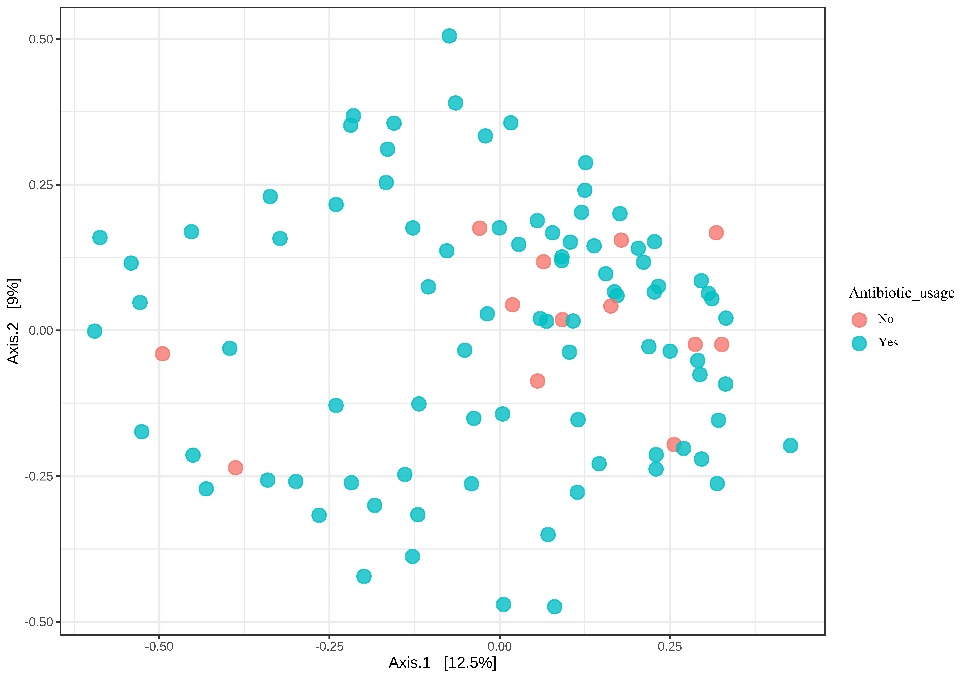


e-Figure 2G. Beta diversity was assessed by PERMANOVA based on Bray-Curtis distances using principal coordinate analysis (PCoA) between patients using antibiotics or not using antibiotics. There is no difference between these two groups (p = 0.552).


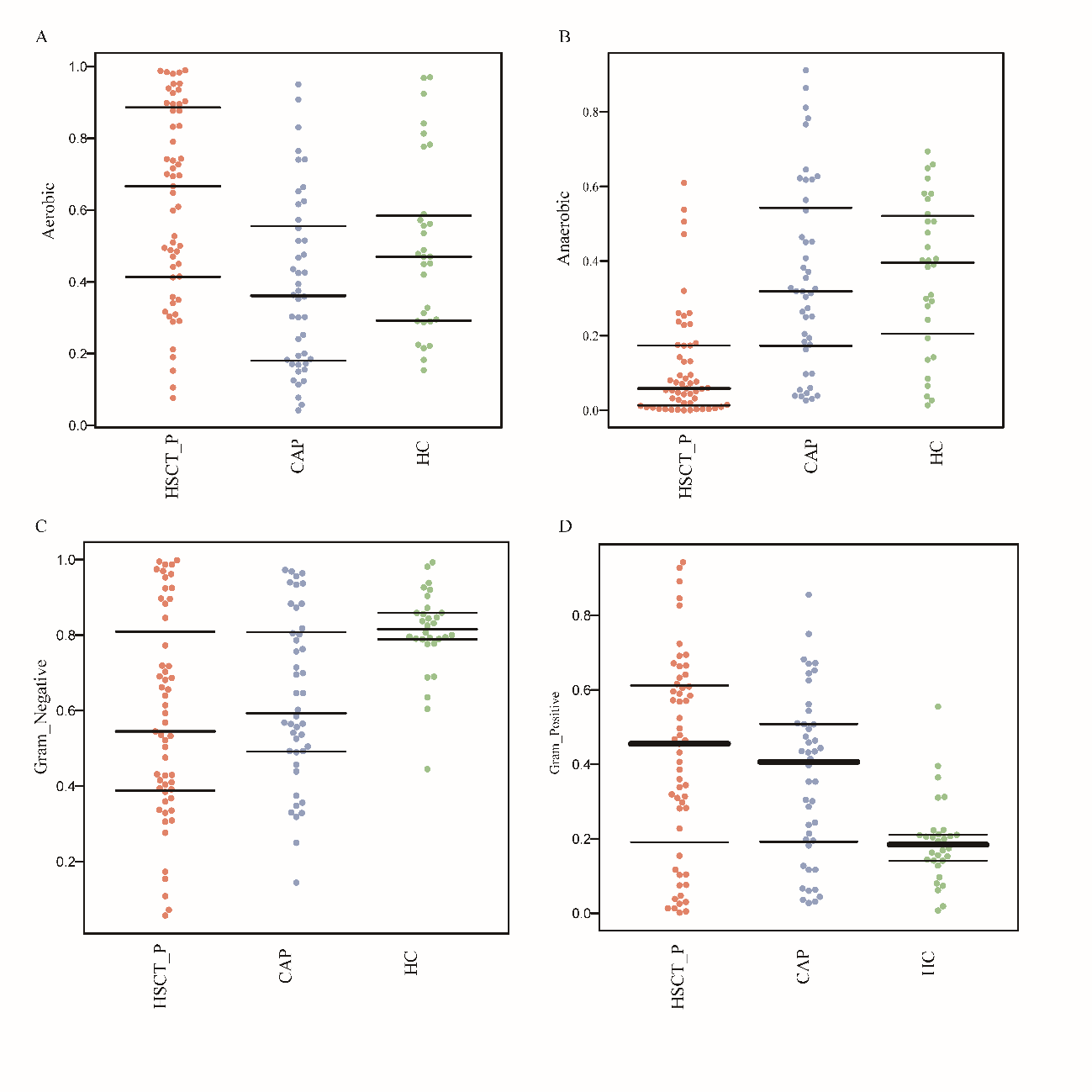


E.

| Phenotype | Group p-value | HSCT_vs_CAP | HSCT_vs_HC | CAP_vs_HC |
| --- | --- | --- | --- | --- |
| Aerobic | 0.0001922839 | 0.0000683 | 0.0364303 | 0.0602027 |
| Anaerobic | 2.599205e-09 | 2.562725e-07 | 4.642226e-07 | 6.976786e-01 |
| Gram_Negative | 0.0003584132 | 0.361815290 | 0.000666973 | 0.001227283 |
| Gram_Positive | 0.0003584132 | 0.361815290 | 0.000666973 | 0.001227283 |

e-Figure 3. Microbiome phenotypes predicted by BugBase. Comparation of microbiome phenotypes including, aerobic (A), anaerobic (B), Gram_negative (C), and Gram_positive (D) among groups. Kruskal-Wallis Test and pairwise Mann-Whitney-Wilcoxon Tests were performed. FDR-corrected pairwise p-values were listed (E).


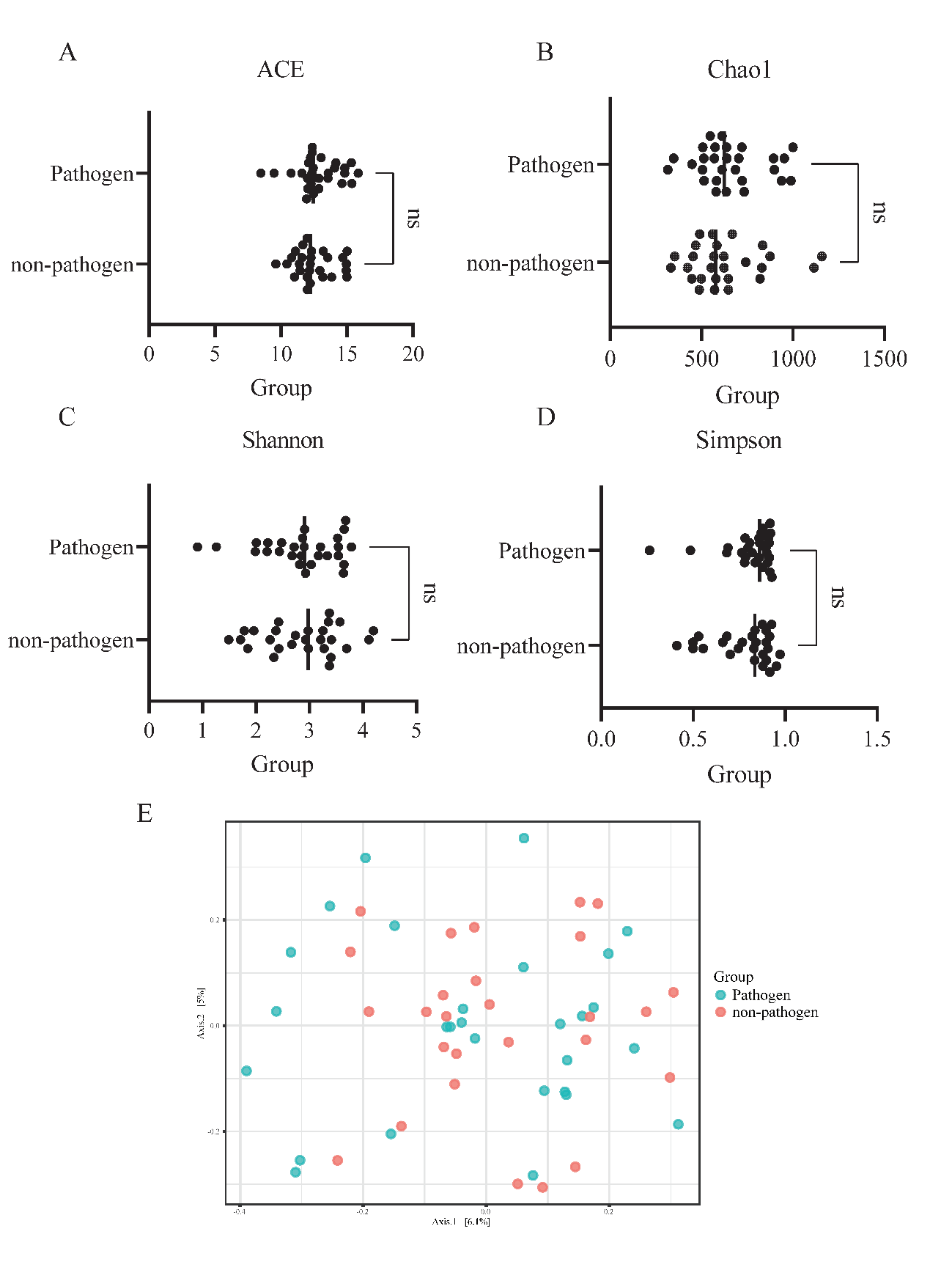


e-Figure 4. Bacterial communities between patients with pathogen and non-pathogen. Alpha diversity was calculated through ACE index (A), Chao1 (B), Shannon (C), Simpson (D). ns, no significant differences were observed between the groups. Beta diversity was evaluated based on jaccard distance.


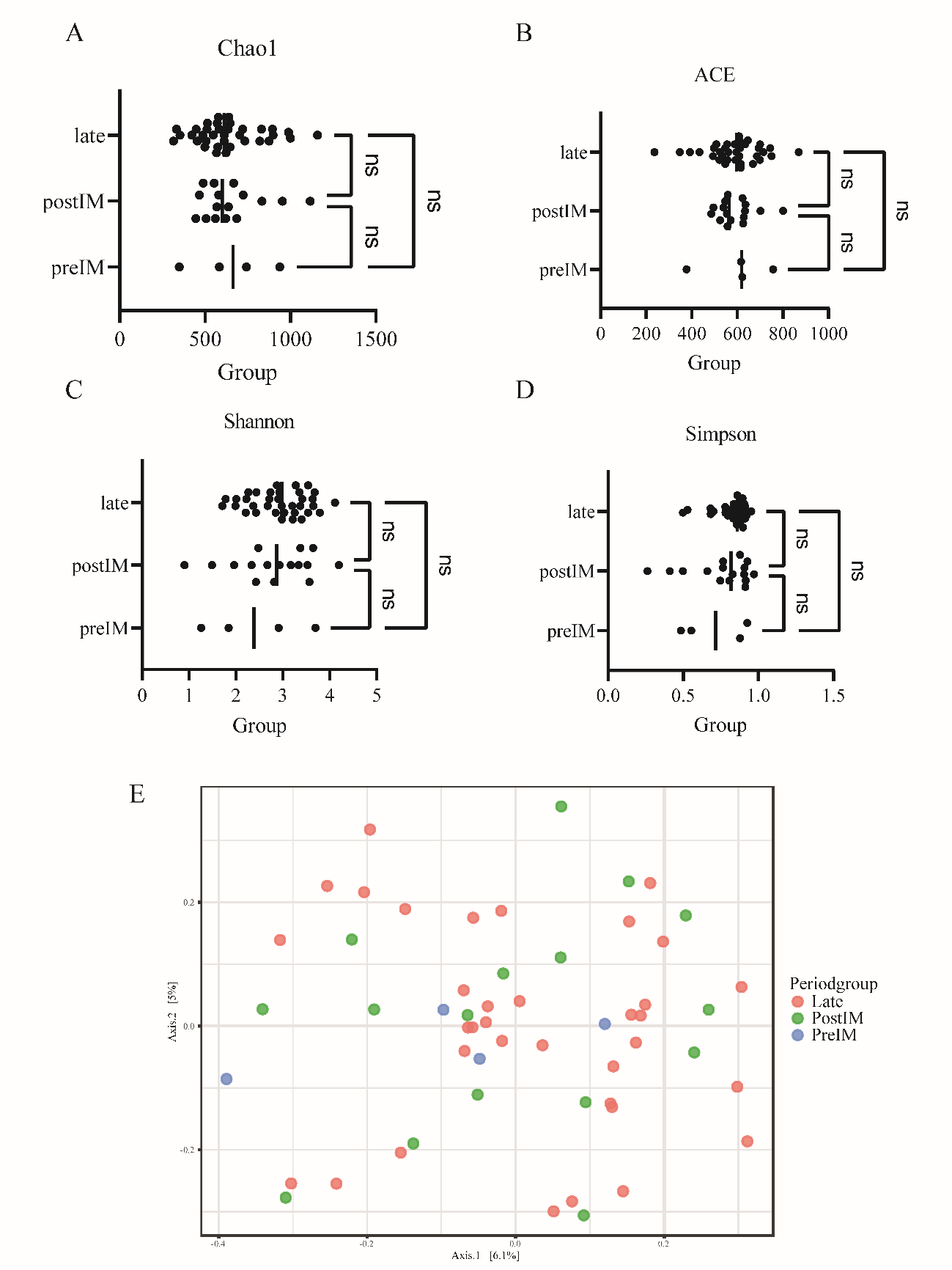


e-Figure 5. Bacterial communities among patients in different periods. Alpha diversity was calculated through ACE index (A), Chao1 (B), Shannon (C), Simpson (D). ns, no significant differences were observed between the groups. Beta diversity was evaluated based on jaccard distance.


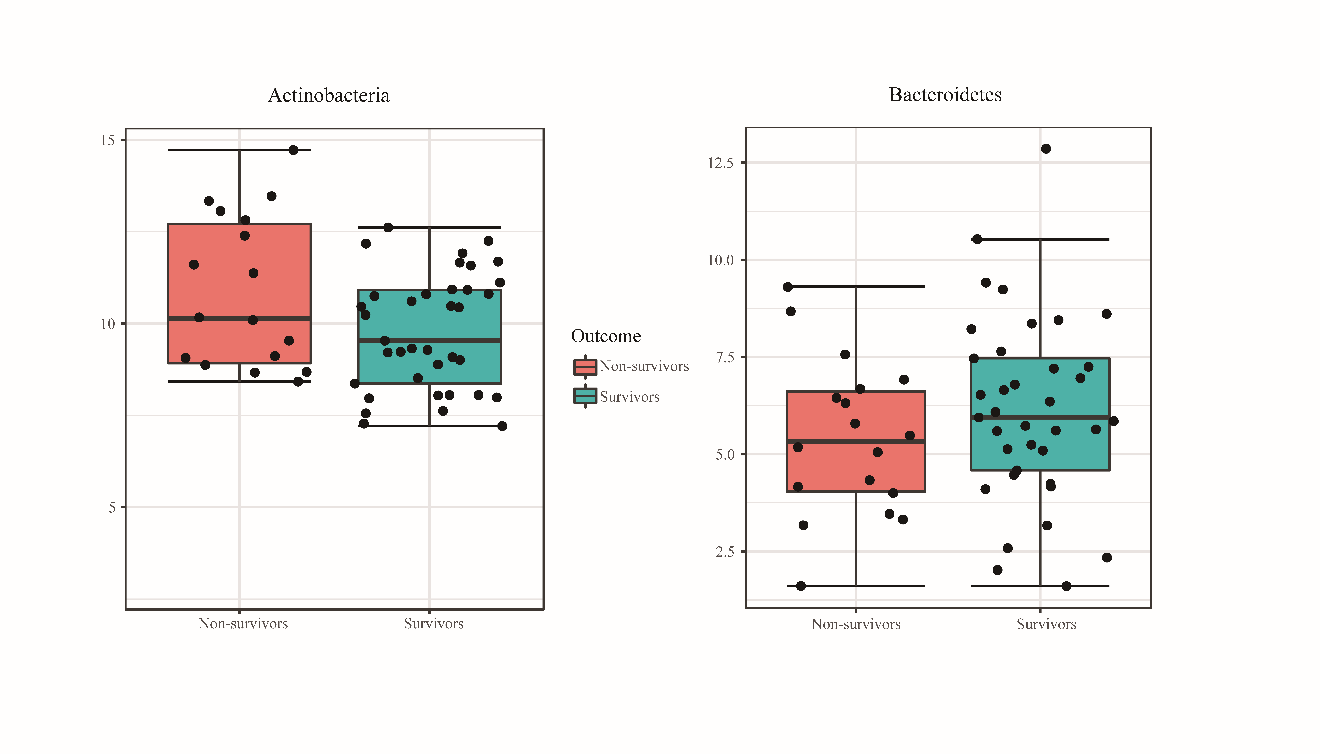


e-Figure 6. Comparison between non-survivors and survivors at phylum level. The abundance of Actinobacteria was richer in non-survivors (*p* = 3.1174E-4) and the Bacteroidetes were decreased in non-survivors (*p* = 0.005).


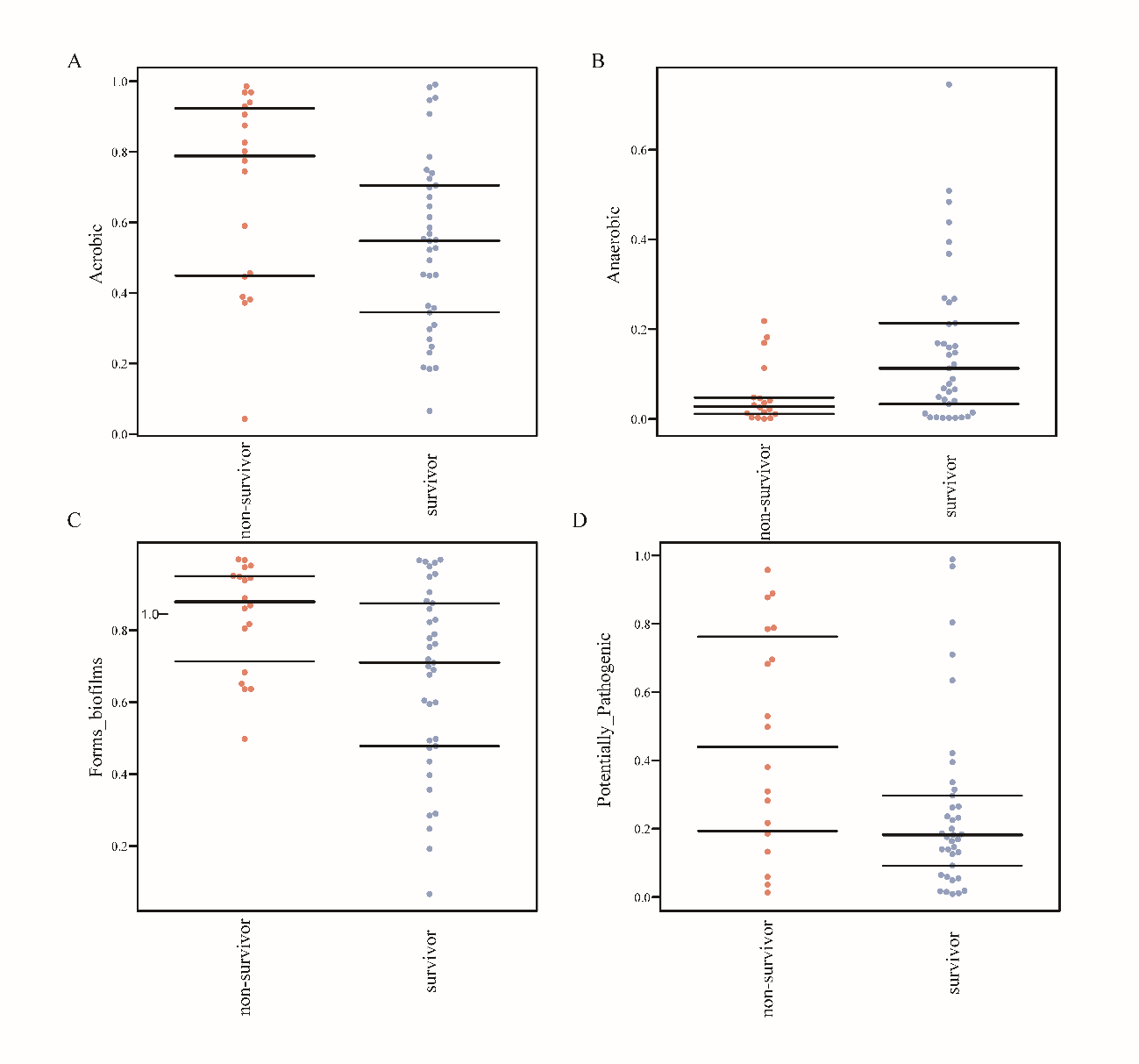


E.

| Phenotype | Group p-value |
| --- | --- |
| Aerobic | 0.04096752 |
| Anaerobic | 0.01645525 |
| Forms_biofilms | 0.01822455 |
| Potentially_Pathogenic | 0.025757 |

e-Figure 7. Microbiome phenotypes of non-survivors and survivors predicted by BugBase. Comparation of microbiome phenotypes including, aerobic (A), anaerobic (B), forms_biofilms (C), and potentially_Pathogenic (D) between these two groups. Mann-Whitney-Wilcoxon Tests were performed. FDR-corrected pairwise p-values were listed (E).
